# UBA2: the gatekeeper of the sumoylation pathway and its multifaceted roles in human diseases and therapeutic prospects

**DOI:** 10.1007/s10238-026-02239-8

**Published:** 2026-06-29

**Authors:** Yi Liu, Qi Deng, Yang Qu, Minming Yi, Taiqiang Wang, Xuan Li, Rong Zhang, Yongkang Wu

**Affiliations:** 1 West China Hospital Sichuan University Jintang Hospital. Jintang First People’s Hospital, Chengdu, 610400 China; 2 Sichuan Jiaotong Hospital, Chengdu, 611730 China; 3 Department of Laboratory Medicine, West China Hospital, Sichuan University, Chengdu, 610041 China

**Keywords:** UBA2, SUMOylation, Developmental malformations, Targeted therapy, Small molecule inhibitors

## Abstract

Ubiquitin-like modifier-activating enzyme 2 (UBA2), the core catalytic subunit of the ubiquitin-activating enzyme (E1) for the small ubiquitin-like modifier (SUMO)ylation pathway, is the “gatekeeper” determining cellular SUMOylation levels. However, a systematic, cross-disease integration of its pathogenic mechanisms and translational value in human diseases is currently lacking. In this review, a disease taxonomy framework was used for the first time to systematically elucidate the multidimensional pathogenic mechanisms of UBA2 in tumors, developmental malformations, autoimmune diseases, and infectious diseases. Research indicates that functional dysregulation of UBA2 is a central node connecting diverse diseases: Driving malignant progression and therapy resistance in tumors, causing congenital malformations during development, acting as a pathogenic factor or autoantigen in autoimmunity, and being targeted by various pathogens for immune evasion. Based on its pivotal role, UBA2 has emerged as an important prognostic biomarker and therapeutic target, with related inhibitors demonstrating promise in preclinical studies. This article further elucidates the strategies and challenges in the clinical translation of precision therapies targeting UBA2, providing a theoretical foundation and translational roadmap.

## Introduction

Small ubiquitin-like modifier (SUMO)ylation is a crucial post-translational modification widely involved in gene expression regulation, genomic stability maintenance, and stress responses. The initiation of this pathway depends on a conserved E1 activating enzyme heterodimer, UBA2/SAE1, in which ubiquitin-like modifier-activating enzyme 2 (UBA2) acts as the catalytic subunit responsible for the ATP-dependent activation of SUMO, designating it as the “gatekeeper” of the SUMOylation cascade [1, 2]. Recent studies have revealed that UBA2 functions extend far beyond its basic enzymatic role, and dysregulation of its expression and activity is a common molecular basis linking multiple human diseases. UBA2 overexpression in tumors drives oncogenic signaling pathways and promotes proliferation and therapy resistance through aberrant SUMOylation [3]; its loss-of-function leads to congenital developmental malformation syndromes [4], can serve as an autoantigen in autoimmune diseases, and is also a key target for pathogens, such as human immunodeficiency virus type 1 (HIV-1), to evade host immunity [5]. These findings point to a central question: How does UBA2 translate its fundamental catalytic function into specific pathogenic mechanisms across diverse pathological contexts?

Addressing this systemic question is crucial for understanding the global pathophysiological significance of SUMOylation and for developing cross-disease targeting strategies. However, current knowledge remains fragmented, lacking an integrated analysis based on disease taxonomy. Therefore, this review aimed to systematically elucidate the molecular regulatory mechanisms of UBA2 and, for the first time, to integrate its multidimensional pathogenic roles in tumors, developmental defects, autoimmune, and infectious diseases. Building on this foundation, we will investigate the clinical translation prospects and challenges of biomarkers and therapeutic strategies targeting UBA2 (for instance, SUMO E1 inhibitors), thereby providing a theoretical framework and an actionable roadmap for precision medicine targeting this key “gatekeeper.”

## Molecular structure and function of uba2: the initiating hub of the sumoylation cascade

### Molecular definition and complex assembly

**Fig. 1:**
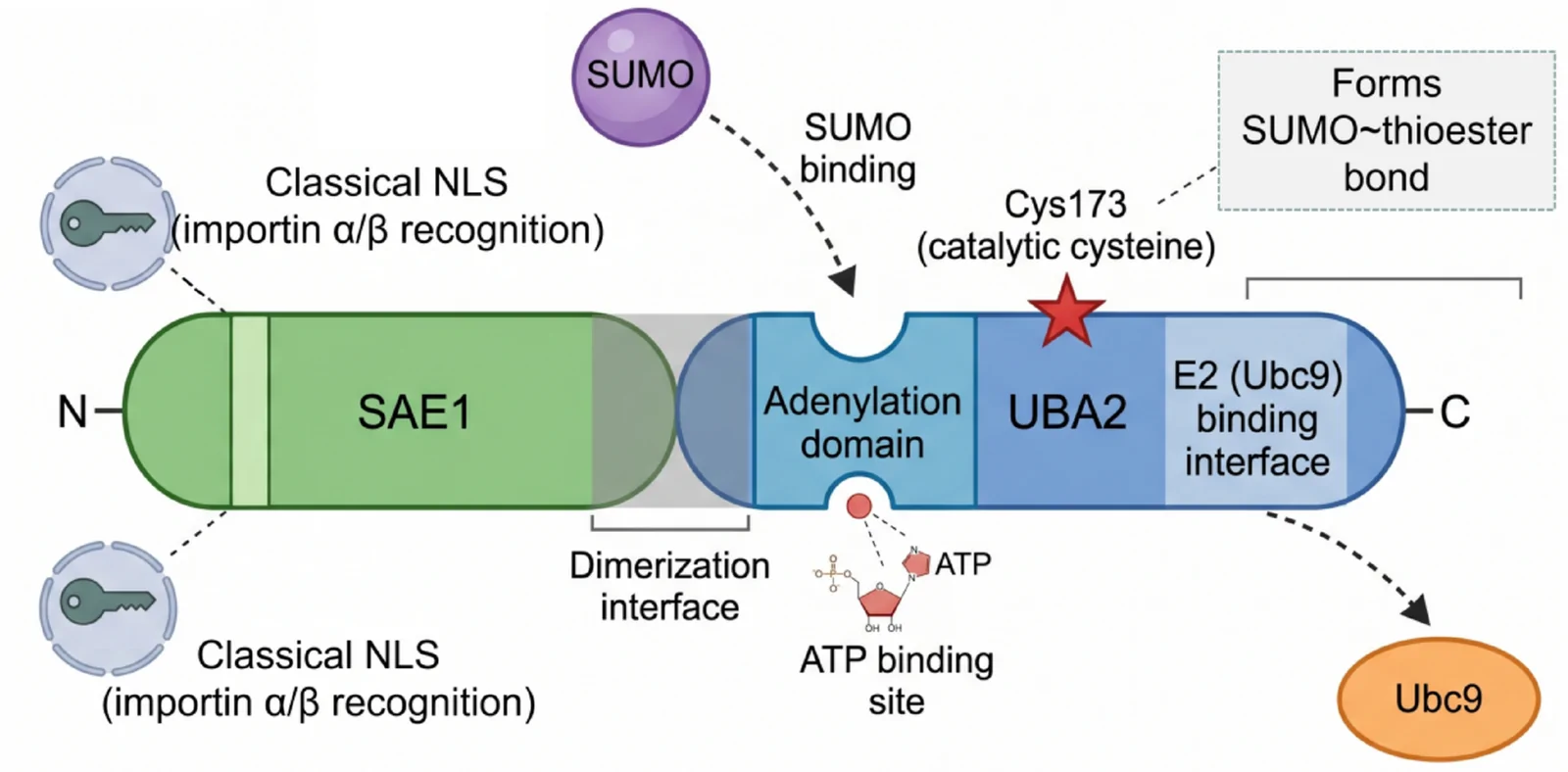
Domain architecture and catalytic core of the UBA2/SAE1 heterodimer. Schematic of the SUMO E1 activating enzyme heterodimer, comprising UBA2 and SAE1. Key features include the ATP-binding site, nuclear localization signal (NLS), dimerization interface, and the catalytic cysteine (Cys173, red star) on UBA2, which forms the SUMO-thioester bond. The diagram highlights SUMO binding and the interaction interface with the E2 enzyme Ubc9, illustrating the initial step of SUMO activation

UBA2, also known as SUMO-activating enzyme subunit 2 (SAE2), forms the sole SUMO-specific E1 activating enzyme heterodimer (UBA2/SAE1 (SUMO-activating enzyme subunit 1)) in mammalian cells together with SAE1 [6]. The identification of this complex was a milestone in the SUMO field, establishing SUMOylation as a crucial post-translational modification pathway distinct from ubiquitination [2]. Its homologs in yeast, Aos1p/Uba2p, are essential for the activation of Smt3p (yeast SUMO), highlighting the evolutionary conservation of this pathway [1]. The UBA2 protein contains multiple functional domains, including a conserved adenylation domain responsible for binding ATP and SUMO, a critical catalytic cysteine residue (Cys173 in humans) for forming the SUMO-thioester intermediate, and a specific interface for interacting with the ubiquitin-conjugating enzyme (E2) Ubc9 [7]. Structural biology studies, by elucidating its crystal structure, have revealed the conformational changes and molecular recognition mechanisms involved in SUMO transfer from UBA2 to Ubc9 [8]. The UBA2/SAE1 heterodimer comprises multiple functional domains, including the adenylation domain, the catalytic cysteine (Cys173), and the E2-binding interface (Fig. 1), which collectively coordinate the binding of ATP and SUMO to initiate the activation cascade.

### Catalytic mechanism and enzymatic properties

**Fig. 2:**
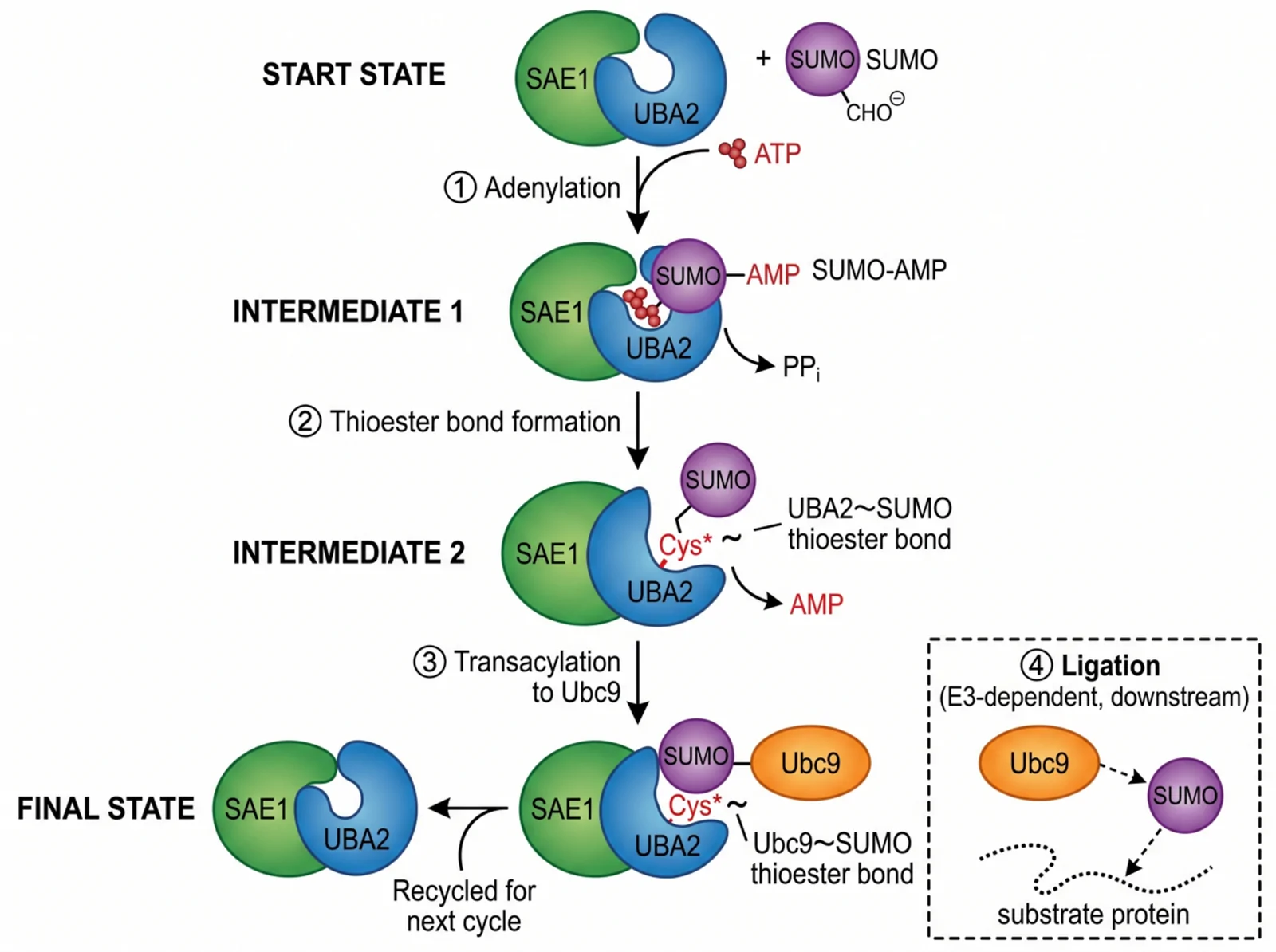
The two-step catalytic cascade of SUMO activation and transfer. Schematic diagram of the SUMO activation and conjugation mechanism catalyzed by the UBA2/SAE1 heterodimer. Key steps include: (1) Adenylation: ATP-dependent formation of a SUMO-adenylate intermediate. (2) Thioester bond formation: Transfer of activated SUMO to the catalytic cysteine (Cys173) of UBA2. (3) Transacylation: Relocation of the SUMO moiety from UBA2 to the E2 conjugating enzyme Ubc9. (4) Ligation: Covalent attachment of SUMO to a lysine residue on the target protein, a step that can be enhanced by E3 ligases. This cascade highlights UBA2’s central role in initiating and coordinating the SUMOylation pathway

The SUMO activation catalyzed by the UBA2/SAE1 complex is a two-step process. First, in the presence of adenosine triphosphate (ATP), the C-terminal carboxyl group of SUMO is adenylated, forming a high-energy SUMO-AMP intermediate. Subsequently, the activated SUMO is transferred to the catalytic cysteine of UBA2 via a thioester bond [9]. Finally, SUMO is transduced from UBA2 to the E2 conjugating enzyme Ubc9 and is covalently attached to specific lysine residues on target proteins with the help of ubiquitin ligases (E3). This two-step catalytic process and the complete cascade of SUMO transfer are illustrated in Fig. 2. Unlike the ubiquitination system, SUMOylation often relies less on E3 ligases, and the UBA2-Ubc9 binary complex is sufficient to catalyze SUMO modification of some substrates [10]. Studies indicate that UBA2 has different affinities for various SUMO paralogs (SUMO1, SUMO2, and SUMO3), with SUMO1 having the highest affinity, which may guide the type of SUMO modification in specific biological processes [11]. Additionally, UBA2 activity can be feedback-regulated through auto-SUMOylation of its C-terminal domain, a finely tuned self-regulatory mechanism [12, 13].

### Subcellular localization and dynamic regulation

The UBA2/SAE1 complex is primarily localized in the nucleus, the main site of action for most of its substrates. Its nuclear import is mediated by the classical importin α/β pathway, which relies on the nuclear localization signal (NLS) on the complex [14]. SUMOylation of UBA2 (catalyzed by Ubc9) improves its binding to nuclear import receptors, thereby promoting nuclear accumulation of the entire complex and forming a positive feedback loop [15]. The activity and stability of UBA2 are dynamically regulated by multiple mechanisms.

Redox regulation: Reactive oxygen species (ROS) can induce the formation of an intermolecular disulfide bond between the catalytic cysteines of UBA2 and Ubc9, reversibly inhibiting the SUMOylation cascade, directly linking cellular oxidative stress status to the post-translational modification network [16].

Cross-talk: Another ubiquitin-like protein, FAT10, can directly bind to and inhibit the activity of the UBA2/SAE1 complex, revealing complex cross-regulation between different ubiquitin-like modification systems [17].

**Fig. 3:**
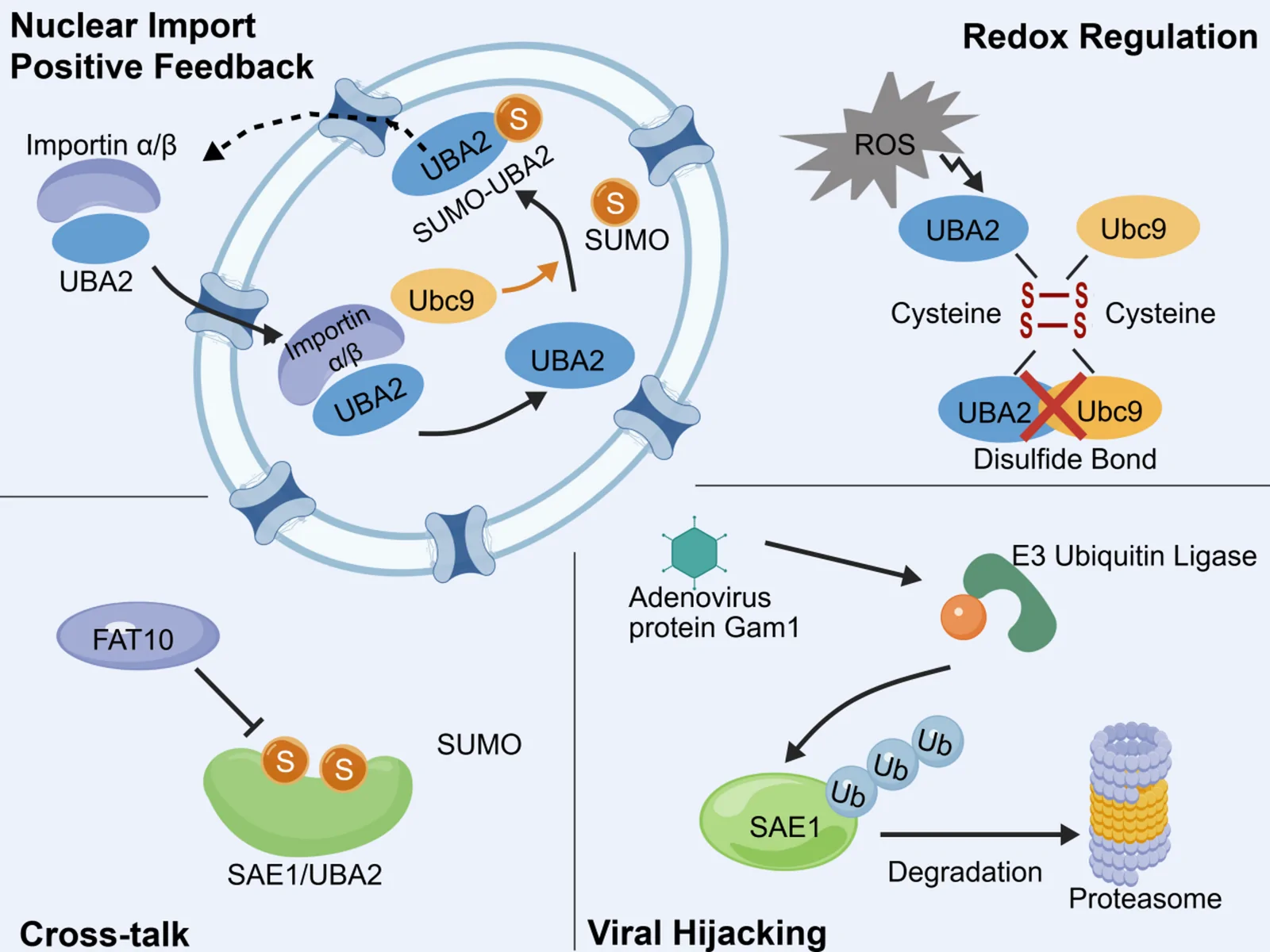
Schematic of UBA2/SAE1 complex dynamic regulation and nuclear import. Schematic diagram of the UBA2/SAE1 heterodimer’s multi-layered regulation and nuclear import. Key mechanisms: (1) Positive feedback: UBA2 SUMOylation enhances binding to importin receptor, promoting nuclear accumulation. (2) Redox regulation: ROS-induced disulfide bonds (S-S) between UBA2 and Ubc9 reversibly inhibit SUMOylation. (3) Cross-talk: FAT10 binds to the complex to suppress its activity. (4) Viral hijacking: Adenovirus Gam1 induces SAE1 degradation, impairing UBA2 function

Viral hijacking: The adenovirus protein Gam1 recruits ubiquitin ligases to target SAE1 for degradation, indirectly disrupting UBA2 function and illustrating a mechanism of viral antagonism of host defense [18]. These dynamic regulatory processes and the nuclear import pathway of the UBA2/SAE1 complex are illustrated in Fig. 3. These regulatory mechanisms ensure that SUMOylation occurs at the appropriate time, place, and intensity. In-depth analysis of UBA2 function has benefited from the development of various sophisticated in vitro reconstitution systems and detection techniques. For example, efficient recombinant SUMOylation protein expression systems [19] and highly sensitive quantitative Förster resonance energy transfer (FRET) kinetic assays [20] have enabled the precise dissection of UBA2’s enzymatic properties, substrate recognition, and dynamic interaction with the E2 enzyme Ubc9 at the molecular level [21].

### Broad physiological functions and developmental roles

Given the large number and functional diversity of SUMOylation substrates, UBA2, as the “master switch,” participates in regulating nearly all core cellular processes.

Embryonic development: UBA2-mediated SUMOylation is crucial for early embryonic development. In mouse models, transient inhibition of SUMOylation during embryogenesis leads to severe craniofacial malformations, syndactyly, and other phenotypes [22]. UBA2 haploinsufficiency is a direct genetic cause of multiple congenital malformation syndromes in humans [4].

Genomic stability: UBA2 ensures normal cell cycle progression by SUMOylating mitotic checkpoint proteins, such as anaphase-promoting complex/cyclosome (APC/C) [23]. Simultaneously, it participates in the DNA damage response, where SUMOylation serves as a key signal for recruiting repair proteins to damage sites [24]. Studies confirm that loss of UBA2 function in yeast and mammalian cells increases DNA damage sensitivity and genomic instability [25]. Additionally, the SUMO modification can be further recognized by the E3 ubiquitin ligase RNF4, which targets SUMOylated proteins for ubiquitylation and degradation—a SUMO-ubiquitin relay that plays a critical role in DNA repair and may offer additional therapeutic opportunities [26].

Stem cells and cell fate: In embryonic stem cells, UBA2 is required for maintaining the pluripotency gene network and suppressing endogenous retroviruses [27]. In the Drosophila hematopoietic system, UBA2 deficiency prevents hematopoietic progenitor cells from entering a proliferative quiescent state, leading to tumor-like overgrowth [28].

Immunity and metabolism: SUMOylation regulates T cell receptor signaling and inflammatory factor production. HIV infection impairs T cell function by downregulating UBA2 [5]. In metabolism, UBA2 is a key upstream node regulating the activity of metabolic enzymes like pyruvate kinase M2 (PKM2) [29]. Furthermore, UBA2 is involved in reproductive biology; supplementing exogenous UBA2 improves the in vitro maturation rate and embryonic development quality of porcine oocytes [30].

## Aberrant expression and pathogenic mechanisms of uba2 in diseases

UBA2 dysfunction, whether through protein overexpression or loss-of-function, is intricately involved in the pathological processes of various human diseases by disrupting the dynamic balance of SUMOylation. UBA2 plays diverse roles in different diseases, ranging from a core driver to a key regulatory node.

### Neoplastic diseases

UBA2 overexpression is a common phenomenon in malignant tumors, where it acts as an “amplifier” for multiple oncogenic signaling pathways by hyperactivating SUMOylation.

**Fig. 4:**
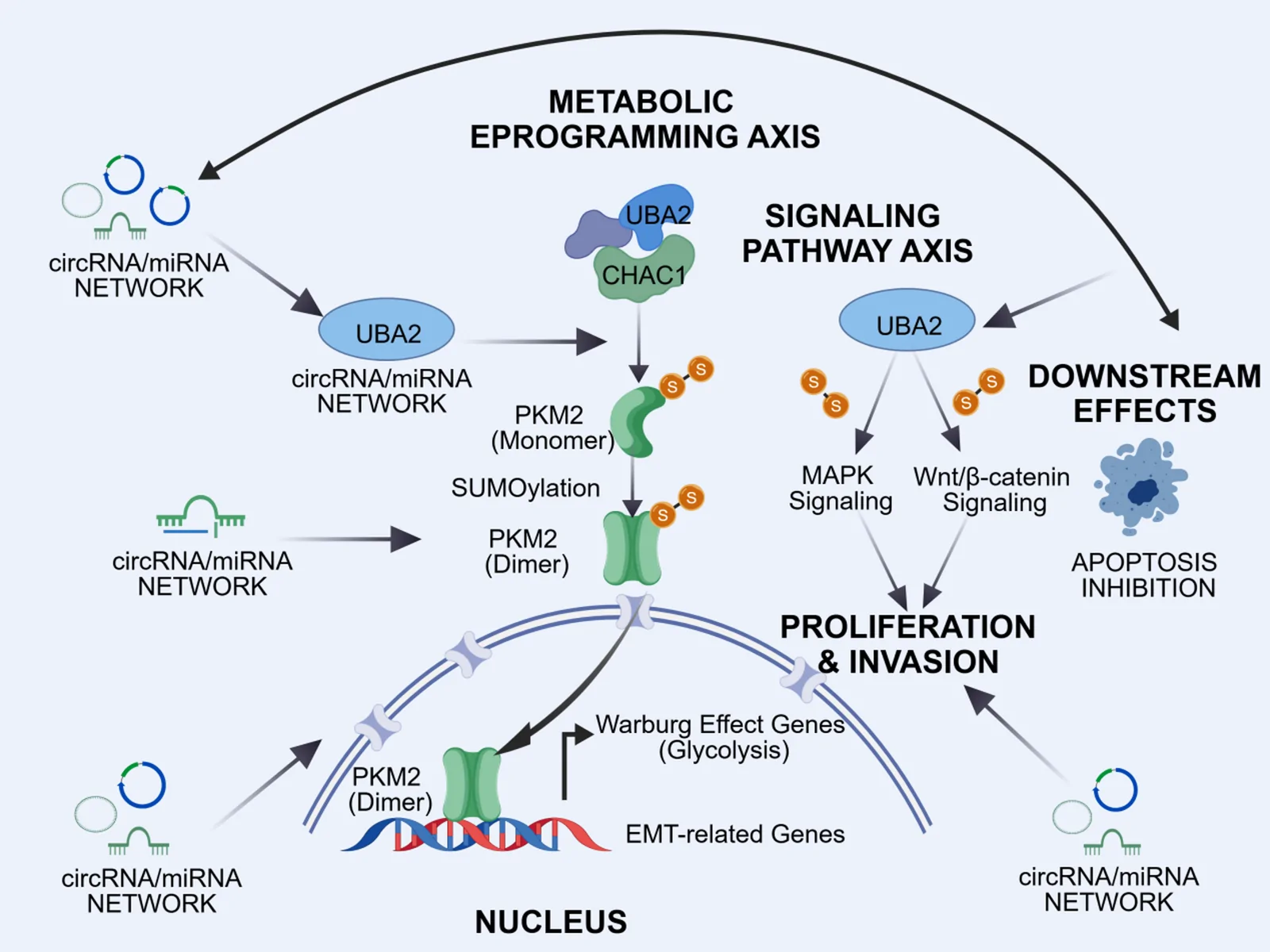
Schematic of UBA2-mediated oncogenic regulatory networks in hepatocellular carcinoma and other gastrointestinal cancers. In hepatocellular carcinoma (HCC), UBA2 activates MAPK signaling, promotes PKM2 SUMOylation (Warburg effect), and upregulates CHAC1. In colorectal and gastric cancers, the Wnt/β‑catenin pathway and circRNA/miRNA regulatory axes are key mediators. These pathways collectively enhance proliferation, invasion, glycolysis, EMT, and survival

In **hepatocellular carcinoma (HCC)**, UBA2 overexpression is closely associated with poor patient prognosis and may drives malignant progression through multidimensional mechanisms [3]. As a SUMO-activating enzyme, UBA2 promotes the SUMOylation of the antioxidant enzyme NAD(P)H: quinone oxidoreductase 1 (NQO1) by regulating the mitogen-activated protein kinase (MAPK) signaling pathway, thereby potentially accelerating HCC cell proliferation, invasion, and migration in vitro [3]. More importantly, UBA2 is central to metabolic reprogramming in HCC. It collaborates with adapter proteins, such as GTP-binding protein 4 (GTPBP4) or ChaC glutathione-specific gamma-glutamylcyclotransferase 1 (CHAC1), to specifically catalyze the SUMOylation of the key glycolytic enzyme PKM2 [31, 32]. SUMOylated PKM2 dimerizes and translocates to the nucleus, activating a series of gene transcriptions that collectively drive the Warburg effect (aerobic glycolysis), thereby providing energy and biosynthetic precursors for rapid tumor cell growth and metastasis, while also promoting epithelial-mesenchymal transition (EMT) [31]. Additionally, UBA2 has been reported to improve the pro-proliferative activity of the transcription factor transcription factor II-I (TFII-I) by SUMOylating it in HCC cells [33]. Further research revealed functional synergy between UBA2 and the E2 conjugating enzyme ubiquitin-conjugating enzyme E2I (UBE2I) in the SUMOylation cascade; the regulatory network formed by both is significantly associated with metastatic propensity and poor prognosis in HCC [34]. Etiologically, aberrant UBA2 expression is also linked to therapy resistance. In HCV-associated HCC, UBA2 downregulation may be associated with tumor cell tolerance to interferon-α (IFN-α) therapy, indicating that its expression level may influence responses to biological treatments [35]. These synergistic effects establish its role as a key oncogenic factor in HCC and are associated with multidrug resistance [36].The multifaceted oncogenic mechanisms orchestrated by UBA2 in hepatocellular carcinoma are summarized in Fig. 4.

In the development and progression of **colorectal cancer (CRC)**, UBA2 has been suggested to play an oncogenic role, potentially contributing to abnormal proliferation by regulating cyclins and the apoptotic network [37]. Its pro‑tumorigenic effect may be partially mediated by sustained activation of the Wnt/β‑catenin signaling pathway [38]. UBA2 expression is finely controlled by a complex, multi-layered regulatory network. The transcription factor forkhead box D3 (FOXD3) inhibits UBA2 translation by inducing miR-133a expression, forming a tumor-suppressive axis [39]. Conversely, the long non‑coding RNA SH3PXD2A‑AS1 has been proposed to competitively bind miR‑330‑5p, which may relieve its inhibition of UBA2, potentially activating the Wnt/β‑catenin pathway and contributing to tumor progression [40]. Furthermore, a set of pro-metastatic miRNAs (miR-424-3p, -503, and − 1292) promotes CRC metastasis by cooperatively downregulating common target proteins, including UBA2 [41]. It is noteworthy that another similarly named but functionally distinct gene— UBA protein 2-like, related to the ubiquitin-proteasome system—is also overexpressed in CRC, and its loss-of-function can inhibit tumor cell proliferation and induce apoptosis [42]. This suggests the existence of multiple aberrant “UBA” family members involved in protein homeostasis regulation in CRC, with UBA2 (the SUMO activator) playing a more central and specific role.

In **gastric cancer (GC)**, UBA2 expression levels are significantly positively correlated with adverse clinical features, including poor tumor differentiation, advanced tumor-node-metastasis (TNM) stage, and vascular invasion [43]. In vitro studies showed that UBA2 knockdown could inhibit GC cell proliferation, migration, and invasion, and could induce cell cycle arrest, a mechanism suggesting involvement of the Wnt/β‑catenin pathway and EMT reversal [43]. The circular RNA circXRCC5 has been reported to upregulate RREB1 by sponging miR‑655‑3p, which then may promote UBA2 transcription in a positive feedback loop, potentially forming a reinforcement circuit that may contribute to GC growth and metastasis [44].

In **non-small cell lung cancer (NSCLC) and lung adenocarcinoma (LUAD)**, UBA2 was found to promote cancer cell proliferation in A549 cells by regulating the expression of cell cycle‑related proteins [45]. A prognostic signature model constructed based on SUMO pathway‑related genes, including UBA2, has been shown to effectively predict survival outcomes in LUAD cohorts and may help distinguish potential chemotherapy responses and immunotherapy microenvironment features, highlighting its value in precision medicine [46]. Further research has confirmed that a two-gene signature based on UBA2 (SAE2) and its partner subunit SAE1 is a robust, independent, poor prognostic factor capable of effectively predicting overall survival in patients with NSCLC [47]. Additionally, the oncoprotein CHAC1 has been reported to act as a molecular bridge, linking UBA2 with PKM2, suggesting a role in promoting PKM2 SUMOylation and nuclear translocation, potentially driving tumor aerobic glycolysis and progression in preclinical models [32]. The hepatocyte growth factor / mesenchymal-epithelial transition factor (HGF/c-Met) signaling pathway can also upregulate UBA2 expression, thereby promoting resistance to RNA-binding protein (RALY) epidermal growth factor receptor (EGFR) tyrosine kinase inhibitors and EMT [48].

In **glioma** progression, aberrantly high UBA2 expression is associated with tumor proliferation, invasion, and remodeling of the immune microenvironment in preclinical glioma models [49]. One of its core mechanisms is has been shown to the SUMOylation of the RNA-binding protein RALY, which may stabilizing and enhancing its function, which subsequently activates the downstream forkhead box D1 (FOXD1) / dickkopf-related protein 1 (DKK1) signaling axis, inducing tumor cells to form highly invasive vasculogenic mimicry [50]. This series of events underscores the critical role of UBA2 in shaping the malignant phenotype of glioma, making it an important prognostic biomarker and therapeutic target [51]. Multi-omics analysis identified UBA2 as a key subtype-specific molecule in low-grade glioma [52].

In **breast cancer**, the antibiotic monensin was found to inhibit breast cancer cell proliferation and migration and induces apoptosis by downregulating UBA2 expression [53]. Notably, breast cancer cells with activated NOTCH1 signaling have been reported to show sensitivity to SUMOylation inhibition, and UBA2 inhibition can impede the growth of such cells in preclinical models [54]. Moreover, a UBA2-programmed cell death 2-like (PDCD2L) fusion gene was identified in breast cancer cells, which may serve as a new therapeutic target [55]. Furthermore, SUMOylation has been suggested to positively regulate the expression of the histone methyltransferase enhancer of zeste homolog 2 (EZH2) via the UBA2-E2F1 axis, which may offer an indirect intervention strategy for targeting EZH2-high breast cancers [56].

In **thyroid cancer (papillary thyroid carcinoma**,** PTC)**, although the expression of multiple components of the SUMOylation pathway is dysregulated, large-scale clinical sample analysis revealed no significant change in UBA2 mRNA levels, indicating that its potential role in PTC pathogenesis may differ from other SUMOylation components [57].

In **renal cell carcinoma (RCC)**, UBA2 was reported to promote tumor cell proliferation and to inhibit apoptosis in RCC cell lines by suppressing the p53 tumor suppressor pathway [58]. In the **clear cell RCC (ccRCC)** subtype, upregulated UBA2 expression is associated with larger tumor volume and higher pathological grade. UBA2 knockdown induces apoptosis in tumor cells by regulating proteins, including mutant p53 and MYC proto-oncogene (c-Myc) [59].

It has been proposed that, in the hypoxic tumor microenvironment of **ovarian cancer (OC)**, UBA2 is recruited by cell-specific molecule 1 (ESM1), potentially serving as a molecular mediator for PKM2 interaction, thereby promoting PKM2 SUMOylation and dimer formation. This may improve glycolytic capacity (Warburg effect) and vasculogenic mimicry, contributing to disease progression as suggested by in vitro and in vivo models [29]. UBA2 expression levels are also associated with chemotherapy resistance in patients with OC, indicating its potential as a predictive biomarker for resistance [60].

In **acute myeloid leukemia (AML)**, a novel oncogenic driver—the UBA2-WTIP (Wilms tumor 1 interacting protein) fusion gene—resulting from the fusion of UBA2 with the WTIP gene, has been identified [61]. This fusion protein has been shown to enhance leukemia cell proliferation in vitro by activating multiple oncogenic signaling pathways, including signal transducer and activator of transcription 3 (STAT3)/ signal transducer and activator of transcription (STAT5), and extracellular signal-regulated kinase 1/2 (ERK1/2), and it may be sensitive to specific drugs, suggesting it could serve as a precision therapy target for this subset of patients [61]. Additionally, combining a SUMO E1 inhibitor with a demethylating agent demonstrates synergistic therapeutic potential in preclinical AML models [62].

A unique monozygotic twin case study of **acute lymphoblastic leukemia (ALL)** indicates that somatic UBA2 gene deletion events may occur before the generation of the well-known oncogenic driver fusion gene ETV6-RUNX1, indicating that UBA2 loss-of-function may act as an early genetic event, creating a susceptible cellular environment for subsequent malignant transformation [63].

In the treatment of **multiple myeloma (MM)**, overexpression of the SUMO E1 enzyme complex containing UBA2 is closely associated with resistance to proteasome inhibitors [64]. Combining the SUMO E1 inhibitor TAK-981 with the proteasome inhibitor carfilzomib can effectively overcome resistance in preclinical models by synergistically inducing cellular stress and apoptosis [64]. Additionally, UBA2 has been identified as a core glutamine metabolism-related gene; its upregulation is associated with poor prognosis in MM, and silencing UBA2 can enhance the efficacy of existing inhibitors [65]. The deubiquitinase ubiquitin-specific protease 5 (USP5) has been suggested to maintain UBA2 stability through its UBA2 domain, potentially stabilizing c‑Maf and contributing to MM progression in preclinical models [66].

In **lymphoma**, for **MYC-driven high-risk B-cell lymphoma**, SUMO E1 inhibitors have been shown to directly inhibit tumor cell growth and can remodel the immune microenvironment, suggesting dual efficacy in preclinical lymphoma models [67]. In **mantle cell lymphoma (MCL)**, the SUMOylation program has been reported to be important for maintaining mitotic fidelity; inhibition of UBA2 was shown to lead to mitotic catastrophe and cell death in MCL cell lines [68]. Furthermore, fusion genes like UBA2-WTIP have been detected in non-Hodgkin B-cell lymphomas [69].

During cisplatin resistance in **laryngeal squamous cell carcinoma(LSCC)**, the oncoprotein TMA7 was reported to promote tumor growth and chemoresistance in laryngeal cancer cells by interacting with UBA2, potentially reducing protective autophagy levels [70].

In **oral squamous cell carcinoma (OSCC)**, upregulated UBA2 expression is associated with poor patient prognosis. A prognostic model based on SUMOylation regulators, including UBA2, can effectively predict patient survival [71].

In **salivary adenoid cystic carcinoma (SACC)**, metastasis-associated miR-155 may target and regulate UBA2, and changes in its expression are associated with the malignant phenotype of the tumor [72]. UBA2 has been identified as an important target in the miRNA regulatory network of this tumor [73].

### Non-neoplastic diseases

Unlike the prevalent overexpression of UBA2 in tumors, the pathogenic mechanisms of UBA2 in non-neoplastic diseases are more diverse, primarily encompassing developmental defects due to gene loss-of-function, autoimmune reactions triggered by its abnormal protein expression or role as an autoantigen, and progression of infectious diseases resulting from its specific targeting by pathogens to subvert host defense [4, 5, 22, 74].

Loss-of-function variants in UBA2 are the genetic basis for numerous **congenital developmental malformation syndromes**. Its heterozygous pathogenic variants (for instance, frameshift and missense mutations) can cause diseases with **split-hand/foot malformation (SHFM)** [75, 76]. Large-scale clinical and molecular studies have established it as a novel causative gene for SHFM [77], and subsequent exome sequencing studies have identified it as a novel susceptibility gene for non-syndromic SHFM [78]. The broader **aplasia cutis congenita with ectrodactyly syndrome (ACCES)** caused by UBA2 haploinsufficiency exhibits a wide phenotypic spectrum, ranging from mild to severe, including skin, limb, and neurological developmental abnormalities [4, 79]. It can also present as atypical spondyloepiphyseal dysplasia, expanding its phenotypic spectrum [80]. These phenotypes closely resemble **19q13.11 microdeletion syndrome**. The various clinical manifestations of this syndrome, particularly aplasia cutis congenita, are believed to be primarily determined by UBA2 haploinsufficiency outside the key deleted region [81, 82]. Moreover, this syndrome shares phenotypic similarities with ectrodactyly-ectodermal dysplasia-clefting syndrome [83]. Animal models confirm the critical role of SUMOylation in development: Transient inhibition of the SUMO E1 enzyme (targeting UBA2/SAE1) during pregnancy leads to complex phenotypes, including craniofacial malformations and syndactyly in offspring mice [22]. Additionally, UBA2 variants are associated with **congenital diaphragmatic hernia (CDH+)** [84] and a multiple malformation syndrome involving skin, eyes, and hip joints [85].

In the genetic study of **congenital limb malformations**, UBA2 has been repeatedly identified as a novel candidate disease-associated gene using exome sequencing and more comprehensive genomic sequencing techniques [86, 87]. Advanced machine learning methods in single-cell tissue-specific gene prioritization also rank UBA2 high among genes associated with congenital limb malformations, confirming its significance [88].

Studies on the molecular mechanism of **Townes-Brocks syndrome (TBS)** have revealed that SUMOylation of the disease-causing protein SALL1 depends on catalysis by the UBA2/SAE1 complex and the E2 enzyme Ubc9, implying that the post-translational modification network involving UBA2 may play a key role in the pathology of this genetic disorder [89].

UBA2 plays a key role in **autoimmune diseases**. In **rheumatoid arthritis (RA)**, the UBA2/SAE1 complex is upregulated in synovial fibroblasts. By mediating PKM2 SUMOylation, it was shown to enhance glycolytic activity, invasiveness, and inflammatory cytokine production in RA synovial fibroblasts, potentially contributing to joint destruction [90]. The role of the SUMOylation pathway (including UBA2) in RA pathogenesis has been systematically summarized in a review [91]. In **dermatomyositis (DM)**, UBA2 (as the SAE2 subunit) is the target of autoimmune attack. Anti-SUMO-activating enzyme (SAE) antibodies are highly specific serological markers for this disease [74, 92]. A positive result not only aids diagnosis but is also associated with an increased risk of skin adverse reactions to hydroxychloroquine in patients [93]. A retrospective analysis highlights the significance of interpreting this antibody result in conjunction with clinical manifestations [94]. Screening for myositis-specific antibodies (including anti-SAE antibodies) in diseases with phenotypes similar to DM, such as idiopathic interstitial pneumonia, is important for differential diagnosis and identifying potential autoimmune features [95]. In **inflammatory bowel disease (IBD)**, impaired SUMOylation pathway function is associated with reduced SUMOylation levels due to downregulated E2 enzyme Ubc9 expression, weakens the protective effect against intestinal epithelial inflammation [96].

**Fig. 5:**
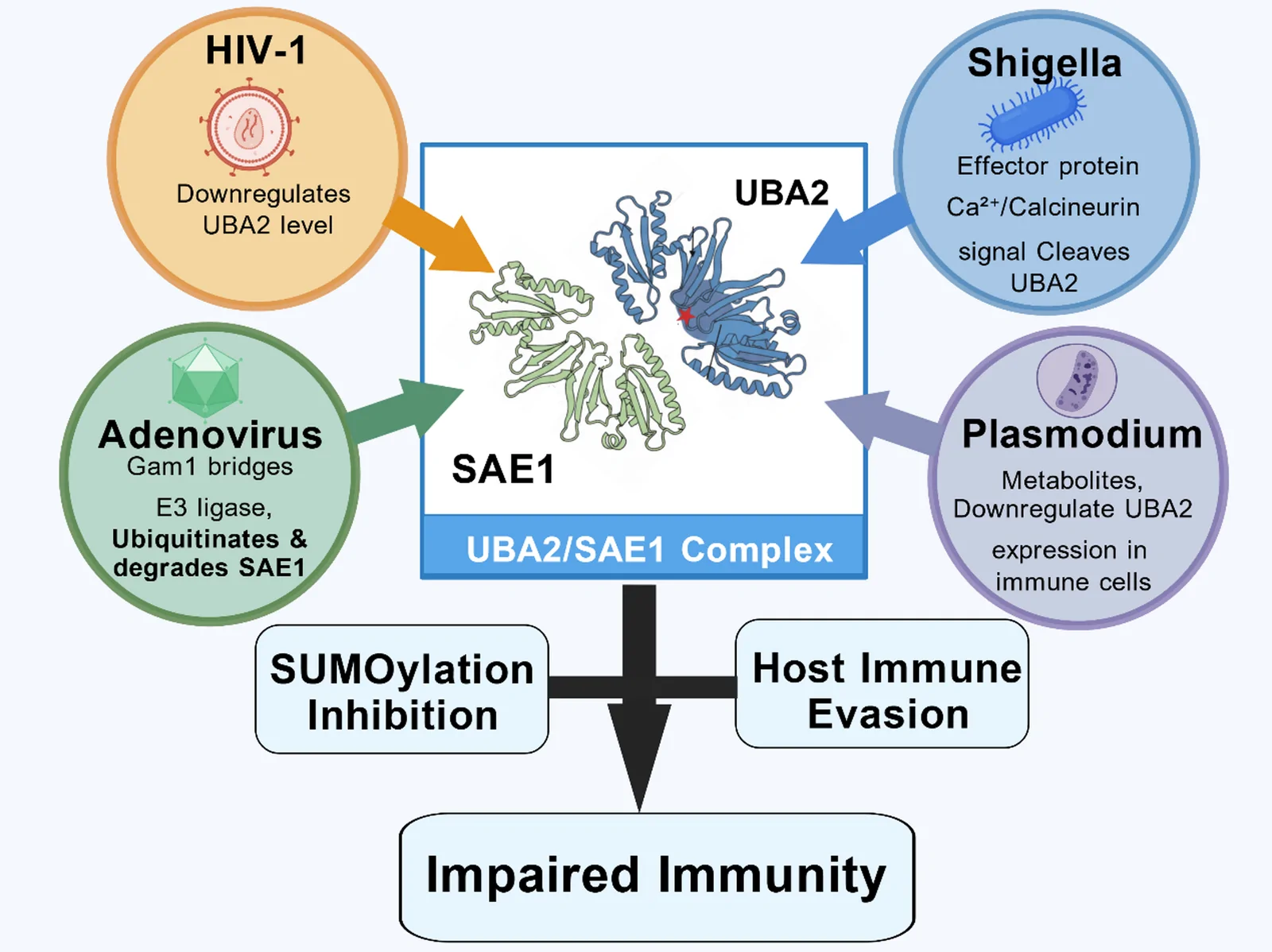
Pathogen-mediated disruption of the host SUMOylation pathway via UBA2/SAE1 targeting. Schematic of convergent strategies used by diverse pathogens to subvert host immunity. HIV-1 downregulates UBA2 protein levels. Shigella effectors cleave UBA2 via a Ca²⁺/calcineurin-dependent mechanism. Adenovirus Gam1 protein recruits an E3 ubiquitin ligase to ubiquitinate and degrade SAE1. Plasmodium metabolites suppress UBA2 expression in immune cells. These distinct inhibitory actions collectively suppress global SUMOylation, leading to impaired host defense

In **infectious diseases**, pathogens strategically disrupt host defense by targeting UBA2. **HIV-1** infection specifically downregulates UBA2 protein levels in host CD4 + T cells, leading to global SUMOylation inhibition, which could be a novel mechanism for reducing host immune system function^6^. The HIV viral protein Vpr can bind to the UBA2 domain of the host DNA repair protein hHR23A, interfering with normal cellular function [97–99]. **Shigella** cleaves UBA2 (SAE2) using a calcium/calcineurin-dependent mechanism, thereby inhibiting the host SUMOylation response to promote its infection [100]. **Plasmodium** utilizes its specific SUMOylation system (containing UBA2 homologs) to cope with oxidative stress [101], and its metabolites can also downregulate UBA2 expression in host immune cells [102]. **Schistosoma** exhibits upregulated expression of SUMO E3 ligases during key developmental stages, suggesting a role for SUMOylation in its life cycle [103]. Regarding **fungal infections**, studies have demonstrated that, under conditions of iron and copper ion availability, various virulence-related proteins, including UBA2, are upregulated in the pathogenic fungus *Cryptococcus neoformans*, closely associated with enhanced fungal pathogenicity. This reveals how metal ion environments can regulate pathogen infectivity by influencing pathways like SUMOylation [104]. Various pathogens, including HIV-1, Shigella, adenovirus, and Plasmodium, use distinct molecular strategies to specifically target and disrupt the function of the host UBA2/SAE1 complex (Fig. 5). This shared mechanism of immune evasion highlights the central role of the SUMOylation pathway in host defense.

In **neurological diseases**, UBA2-mediated SUMOylation dysregulation is involved in pathological processes. In **Alzheimer’s disease**, structural changes in the ubiquitin mutant Ubb + 1 may affect its interaction with the UBA2 domain [105]. UBA2 has been identified as a differentially expressed gene in **multiple sclerosis (MS)** gene expression profiles [106]. Drosophila model studies indicate that the SUMOylation pathway (including UBA2) is involved in regulating polyglutamine protein toxicity related to **spinal and bulbar muscular atrophy (SBMA)** [107]. In an **optic nerve injury** model, mesenchymal stem cell-derived exosomes have been suggested to exert a retinal protective effect, potentially through activation of the UBA2/Wnt/β-catenin pathway [108]. Changes in SUMO-2/3 modification levels after **cerebral ischemia** also involve UBA2 function [109].

Regarding **ophthalmic diseases**, temporal expression changes in SUMOylation enzymes (including UBA2) in a mouse model of **age-related macular degeneration (AMD)** suggest the involvement of this pathway in retinal degenerative disease processes [110]. During **cataract** formation, oxidative stress (for instance, glucose oxidase treatment or UVA irradiation) leads to significant changes in the expression profile of SUMOylation enzymes in lens cells [111]. Differential expression and unique subcellular localization patterns of UBA2 exist across various ocular cell lines, providing a basis for studying its function in eye development and disease [112–114].

In the context of **cardiovascular diseases**, angiotensin II promotes SUMO2/3 modification of RhoGDI1 via Aos1/Uba2 during **hypertensive vascular remodeling**, thereby stabilizing this protein and promoting vascular smooth muscle cell proliferation. This mechanism demonstrates a specific role for SUMOylation in hypertension pathology [115].

In **hepatic ischemia-reperfusion injury**, augmenter of liver regeneration protects mitochondrial function and reduces hepatocyte apoptosis by inhibiting UBA2 expression, thereby reducing SUMOylation of the mitochondrial fission protein Drp1 [116].

In the pursuit of disease-modifying therapies for **cystic fibrosis**, UBA2 was identified via high-throughput functional genomic screening as one of the regulators affecting the processing and membrane localization of the most common mutant, F508del-CFTR protein. Silencing UBA2 was reported to partially restore mutant protein function in cell‑based screens, suggesting a new approach for correcting protein folding defects by modulating SUMOylation and related pathways [117].

In long-term **radiation-related injury** studies, analysis of tissues from patients with bladder urothelial lesions after the Chernobyl accident revealed significant upregulation of ubiquitination and SUMOylation pathway-related proteins, including SUMO1 and Ubc9. This suggests that SUMOylation modifications involving UBA2 may be an adaptive response of cells to persistent genotoxic stress and could be involved in the early stages of radiation carcinogenesis [118].

Basic research in **reproductive medicine and embryonic development** has revealed the role of UBA2 in germ cell health. In porcine oocyte in vitro maturation systems, supplementing UBA2 by elevating SUMO-1 protein levels significantly improves oocyte maturation rate, blastocyst formation rate, and regulates apoptosis-related gene expression, providing a potential strategy to enhance assisted reproductive technology efficiency [30]. Furthermore, in early porcine embryos, DNA damage dynamically regulates the expression of ubiquitination/SUMOylation-related genes, including UBA2, hinting at the vital role of this pathway in maintaining embryonic genome stability [119].

As the initiating engine of SUMOylation pathway, UBA2 functions extend far beyond its direct role in specific diseases; it regulates fundamental biological processes, including cell fate determination and tissue homeostasis maintenance. In **embryonic stem cells**, UBA2 is key to maintaining pluripotency and genomic integrity. It effectively suppresses the expression of endogenous retroviruses by SUMOylating key proteins of the silencing complex, safeguarding stem cell identity [27]. This role extends to **tissue stemness and tumor prevention**. In a Drosophila model, UBA2 maintains the proliferative quiescence of hematopoietic progenitor cells by SUMOylating and stabilizing cell cycle inhibitors. Its loss-of-function leads to over-proliferation and tumor-like transformation of stem cells, demonstrating its intrinsic property as a “guardian” of tissue homeostasis [28]. Regarding **cell life cycle regulation**, related studies indicate that UBA2 may be involved in regulating senescence and death programs [120]. At the **epigenetic level**, UBA2 is an essential factor for maintaining genome-wide gene silencing states, indicating that SUMOylation is an infrastructure supporting higher-order chromatin structure and epigenetic memory [121]. Additionally, in **embryonic development programming**, the SUMOylation pathway involving UBA2 rapidly responds to DNA damage to maintain embryonic genome stability [119] and finely regulates the activity of key cardiac transcription factors, affecting organ formation [122]. These studies indicate that UBA2 is a fundamental molecular switch regulating cell identity, genomic stability, epigenetic landscape, and developmental programs. This explains why its dysfunction leads to widespread and severe pathological consequences and indicates that any global intervention strategy must carefully weigh its potential impact on basic physiological processes.

In summary, aberrant expression and function of UBA2 constitute a common molecular pathological basis widely permeating tumors, developmental defects, autoimmunity, infections, and various systemic diseases (Table 1). Its central position in the SUMOylation pathway makes it a crucial window for understanding disease mechanisms and a highly promising target for developing novel diagnostic and therapeutic strategies. From promoting metabolic reprogramming in cancer cells to ensuring normal embryonic morphogenesis, from regulating immune-inflammatory balance to becoming a target for pathogen attack, the multifaceted functions of UBA2 have left a profound and complex imprint on the landscape of human diseases.

**Table 1:** Aberrant UBA2 Function in Human Diseases: Mechanisms and Clinical Associations

Disease Category	Disease	Core Mechanism of Action	Key Molecular Pathway/Target	Biological Effect	Clinical Association	Refs.
Neoplastic Diseases	Hepatocellular Carcinoma (HCC)	UBA2 overexpression catalyzes SUMOylation of substrates like NQO1, PKM2, and TFII-I, driving metabolic reprogramming.	MAPK pathway; GTPBP4/UBA2/PKM2 axis; CHAC1/UBA2/PKM2 axis.	Promotes proliferation, invasion, migration; induces aerobic glycolysis and EMT.	High expression correlates with poor prognosis and multidrug resistance.	[3, 31–36]
	Colorectal Cancer (CRC)	UBA2 overexpression drives proliferation; regulated by FOXD3/miR-133a inhibition and SH3PXD2A-AS1/miR-330-5p upregulation.	Wnt/β-catenin signaling pathway.	Promotes proliferation, migration, invasion.	High expression promotes progression and is a poor prognostic factor.	[37–41]
	Gastric Cancer (GC)	UBA2 overexpression, regulated by a circXRCC5/miR-655-3p/RREB1 positive feedback loop.	Wnt/β-catenin signaling pathway.	Promotes proliferation, migration, invasion; induces cell cycle arrest.	Expression positively correlates with poor differentiation, advanced stage, and vascular invasion.	[43, 44]
	Non-Small Cell Lung Cancer / Lung Adenocarcinoma (NSCLC/LUAD)	UBA2 overexpression promotes cell cycle progression; CHAC1 bridges UBA2 and PKM2, facilitating its SUMOylation and nuclear translocation.	Cyclins; CHAC1-UBA2-PKM2 axis; HGF/c-Met pathway.	Promotes proliferation; drives glycolysis and EMT; mediates EGFR-TKI resistance.	High expression predicts poor prognosis; SUMO pathway models can predict treatment response.	[32, 45–48]
	Glioma	UBA2 overexpression globally alters the SUMOylation landscape; catalyzes SUMOylation of RALY.	UBA2/RALY/FOXD1/DKK1 axis.	Drives proliferation, invasion, and induces vasculogenic mimicry.	Important prognostic marker and therapeutic target; subtype-specific molecule.	[49–52]
	Breast Cancer	UBA2 expression can be downregulated by monensin; NOTCH1 activation increases sensitivity to SUMOylation inhibition.	NOTCH1 signaling pathway; UBA2-E2F1-EZH2 axis.	UBA2 inhibition impedes growth, induces cell cycle arrest and apoptosis.	UBA2-PDCD2L fusion gene may be a novel target.	[53–56]
	Thyroid Cancer (Papillary) (PTC)	Dysregulated expression of SUMO pathway components, but UBA2 mRNA levels show no significant change.	SUMOylation pathway.	Potential role differs from other SUMOylation components.	UBA2 mRNA expression is not a prognostic marker for PTC.	[57]
	Renal Cell Carcinoma / Clear Cell Renal Cell Carcinoma (RCC/ccRCC)	UBA2 overexpression inhibits the p53 pathway; regulates mutant p53 and c-Myc.	p53 signaling pathway.	Promotes proliferation and inhibits apoptosis.	High expression correlates with worse survival, larger tumor volume, and higher grade.	[58, 59]
	Ovarian Cancer (OC)	Under hypoxia, UBA2 is recruited by ESM1 to mediate PKM2 SUMOylation.	ESM1/UBA2/PKM2 axis.	Enhances glycolytic and vasculogenic mimicry capacity.	Expression level correlates with chemotherapy resistance.	[29, 60]
	Acute Myeloid Leukemia (AML)	UBA2 fuses with the WTIP gene, producing the oncogenic UBA2-WTIP fusion protein.	STAT3, STAT5, ERK1/2 pathways.	Significantly enhances leukemia cell proliferation.	Novel oncogenic driver and precision therapy target.	[61, 62]
	Acute Lymphoblastic Leukemia (ALL)	Somatic UBA2 gene deletion may serve as an early event, creating a susceptible environment.	SUMOylation/DNA repair pathways.	UBA2 loss-of-function may create conditions for malignant transformation.	UBA2 deletion may be a marker of early genetic events in ALL.	[63]
	Multiple Myeloma (MM)	High expression of the SUMO E1 complex (containing UBA2) correlates with drug resistance; it is a core glutamine metabolism gene.	Proteasome pathway; glutamine metabolism pathway.	Drives disease progression; UBA2 silencing sensitizes cells.	High expression correlates with poor prognosis; SUMO E1 inhibitors can overcome resistance.	[64–66]
	Lymphoma (High-risk B-cell lymphoma, Mantle Cell Lymphoma (MCL))	MYC-driven tumors are sensitive to SUMO inhibition; SUMOylation maintains mitotic fidelity.	MYC signaling pathway.	UBA2 inhibition causes mitotic catastrophe and cell death; remodels the immune microenvironment.	SUMO E1 inhibitors exhibit dual efficacy: direct anti-tumor and immunomodulatory.	[67–69]
	Laryngeal Squamous Cell Carcinoma (LSCC)	Oncoprotein TMA7 interacts with UBA2, inhibiting protective autophagy.	TMA7/UBA2/PI3K axis.	Promotes tumor growth and cisplatin resistance.	The TMA7/UBA2 interaction may be a target for reversing resistance.	[70]
	Oral Squamous Cell Carcinoma (OSCC)	Upregulated UBA2 expression.	SUMOylation pathway.	Promotes malignant progression.	High expression correlates with poor prognosis; SUMO regulator-based models can predict survival.	[71]
	Salivary Adenoid Cystic Carcinoma (SACC)	Metastasis-associated miR-155 may target UBA2.	miRNA regulatory network (e.g., miR-155).	Involved in tumor malignant progression.	UBA2 is an important target in the miRNA regulatory network.	[72, 73]
Non-Neoplastic Diseases	Split-Hand/Foot Malformation (SHFM)	UBA2 loss-of-function variants cause haploinsufficiency.	SUMOylation pathway.	Limb development abnormalities.	UBA2 is a novel causative/susceptibility gene for SHFM.	[75– [78, 86, 87]
	Aplasia Cutis Congenita with Ectrodactyly Syndrome (ACCES)	UBA2 haploinsufficiency (loss-of-function or missense variants).	SUMOylation pathway.	Broad phenotypic spectrum: skin, limb, neurodevelopmental abnormalities, etc.	UBA2 variants are the genetic cause of ACCES; high phenotypic heterogeneity.	[4, 79, 80, 85]
	19q13.11 Microdeletion Syndrome	Haploinsufficiency of UBA2 (outside the deletion region) is the core pathogenic factor.	SUMOylation pathway.	Multiple abnormalities including aplasia cutis congenita.	UBA2 haploinsufficiency dictates its main clinical manifestations.	[81–83]
	Congenital Diaphragmatic Hernia (CDH+)	UBA2 haploinsufficiency may interfere with diaphragm development.	SUMOylation pathway.	Diaphragm development abnormality.	UBA2 identified as a candidate disease gene for CDH+.	[84]
	Townes-Brocks Syndrome (TBS)	SUMOylation of the disease-causing protein SALL1 depends on UBA2/SAE1 and Ubc9.	SUMOylation pathway.	Limb, ear, anal malformations.	UBA2-involved SUMOylation network may play a key role in TBS pathology.	[89]
	Rheumatoid Arthritis (RA)	Upregulated UBA2/SAE1 complex in synovial fibroblasts mediates PKM2 SUMOylation.	PKM2-related glycolytic pathway.	Enhances cell glycolysis, invasiveness, and inflammatory responses.	UBA2/SAE1 is a potential therapeutic target for RA.	[90, 91]
	Dermatomyositis (DM)	UBA2 (SAE2 subunit) acts as an autoantigen, generating anti-SAE antibodies.	Autoimmune response.	Elicits specific autoimmune response.	Anti-SAE antibody is a highly specific diagnostic marker for DM and predicts hydroxychloroquine skin adverse reaction risk.	[74, 92–95]
	Inflammatory Bowel Disease (IBD)	Impaired SUMOylation pathway function; downregulated Ubc9 expression reduces global SUMOylation levels.	SUMOylation pathway (particularly Akt1 SUMOylation).	Weakens protective effect against intestinal epithelial inflammation.	Reduced SUMOylation levels correlate with disease severity.	[96]
	HIV-1 Infection	Virus downregulates UBA2 protein levels in CD4 + T cells; viral protein Vpr binds the UBA2 domain of hHR23A.	Global SUMOylation inhibition; DNA repair interference.	Impairs T cell function, potentially depleting the immune system.	Reveals a novel mechanism for HIV-mediated host immune impairment.	[5, 97–99]
	Shigella Infection	Bacteria cleave UBA2 (SAE2) via a Ca²⁺/calcineurin-dependent mechanism.	SUMOylation pathway inhibition.	Inhibits host SUMOylation response, promoting infection.	Mechanism of pathogen evasion of host defense.	[100]
	Malaria	Plasmodium utilizes its own SUMO system (containing UBA2 homolog) to cope with stress; its metabolites downregulate UBA2 in host immune cells.	Oxidative stress response pathway; host ubiquitination/SUMOylation system.	Favors parasite survival; interferes with host immunity.	Reveals a new layer of interaction between parasite and host SUMOylation systems.	[101, 102]
	Schistosomiasis	Schistosome upregulates SUMO E3 ligase expression during key developmental stages.	Parasite SUMOylation system.	May play an important role in parasite developmental transition.	Suggests SUMOylation as a potential target for anti-parasitic therapy.	[103]
	Fungal Infection (Cryptococcus neoformans)	Under iron/copper ion conditions, upregulation of virulence-related proteins (including UBA2) in fungi.	Metal ion stress response pathway.	Enhances fungal pathogenicity.	Reveals how environmental metal ions regulate pathogen infectivity via pathways like SUMOylation.	[104]
	Alzheimer’s Disease (AD)	Structural changes in ubiquitin mutant Ubb + 1 may affect its interaction with the UBA2 domain.	Ubiquitin-proteasome system/protein interaction.	May interfere with ubiquitin-related protein function.	Suggests UBA2-related pathways may be involved in neurodegeneration.	[105]
	Multiple Sclerosis (MS)	UBA2 identified as a differentially expressed gene.	PARK7 interactome/cell apoptosis and acetylation pathways.	May play a role in autoimmune response.	First report of UBA2 as a differentially expressed gene in MS.	[106]
	Spinal and Bulbar Muscular Atrophy (SBMA)	Drosophila model suggests SUMOylation pathway involvement in regulating polyglutamine protein toxicity.	SUMOylation/ubiquitin-proteasome pathway.	Exacerbates neuronal degeneration caused by polyglutamine protein.	Suggests SUMOylation as a potential intervention target.	[107]
	Age-related Macular Degeneration (AMD)	Temporal changes in SUMOylating enzyme (including UBA2) expression in a mouse model.	Oxidative stress response pathway.	Participates in retinal degeneration process.	Suggests dynamic regulation of the SUMOylation pathway in AMD pathogenesis.	[110]
	Cataract	Oxidative stress causes significant changes in the SUMOylating enzyme expression profile in lens cells.	Oxidative stress response pathway.	Participates in lens opacification process.	Suggests SUMOylation abnormalities may be associated with oxidative stress-related eye diseases.	[111]
	Hypertensive Vascular Remodeling	Angiotensin II promotes SUMO2/3 modification of RhoGDI1 via Aos1/Uba2.	Rho signaling pathway.	Stabilizes RhoGDI1, promoting vascular smooth muscle cell proliferation.	Reveals a specific role of SUMOylation in hypertension pathology.	[115]
	Hepatic Ischemia-Reperfusion Injury (IRI)	Augmenter of liver regeneration reduces UBA2 expression by inhibiting YY1, decreasing Drp1 SUMOylation.	Mitochondrial dynamics pathway.	Protects mitochondrial function, reduces hepatocyte apoptosis.	Provides a new intervention target idea for IRI prevention/treatment.	[116]
	Cystic Fibrosis (CF)	UBA2 identified as a regulator of the processing of the most common mutant, F508del-CFTR protein.	Protein folding and degradation pathway.	UBA2 silencing can partially rescue mutant protein function.	Suggests a new approach to correct protein folding defects by modulating SUMOylation and related pathways.	[117]
	Radiation-Related Injury	Upregulated ubiquitination/SUMOylation pathway proteins in bladder urothelium of patients with long-term low-dose radiation exposure.	Ubiquitination/SUMOylation stress response.	May be an adaptive cellular response to genotoxic stress.	Suggests this pathway may be involved in early radiation carcinogenesis.	[118]
	Reproductive Medicine/Embryonic Developmental Abnormalities	UBA2 supplementation improves porcine oocyte in vitro maturation rate and embryo quality; DNA damage regulates related gene expression in embryos.	SUMOylation pathway; DNA damage response.	Improves oocyte maturation, reduces apoptosis; maintains embryonic genome stability.	Provides potential strategies for improving ART efficiency and assessing embryo health.	[30, 119]

## Clinical translation: from biomarkers to targeted therapy

**Fig. 6:**
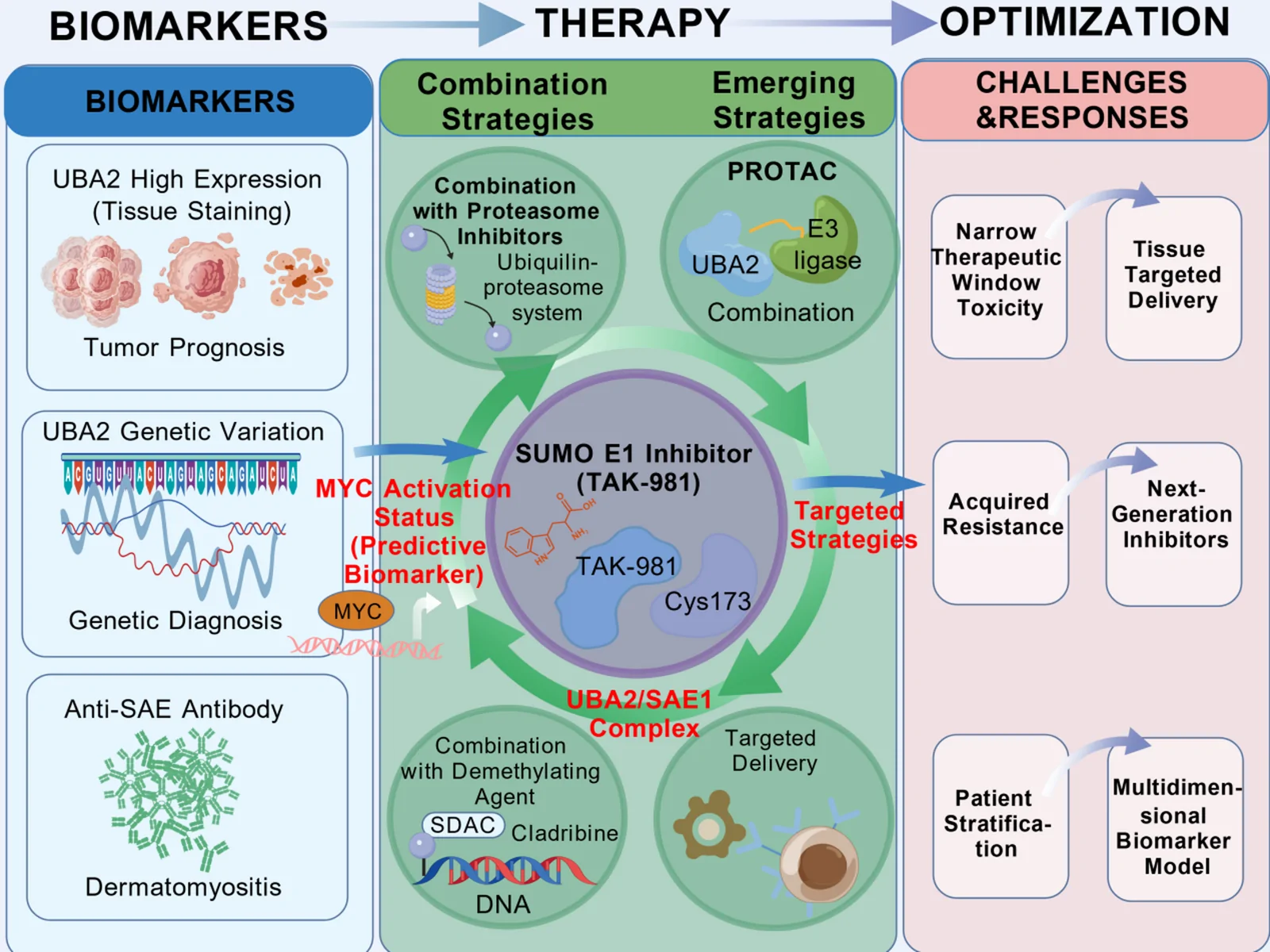
Clinical translation pathway of UBA2/SAE1 targeting. Schematic overview spanning biomarker applications, therapeutic strategies, and future challenges. Biomarkers: UBA2 high expression (tumor prognosis), genetic variation (hereditary diagnosis), anti-SAE antibody (dermatomyositis), and predictive MYC activation status. Therapy: Central mechanism of SUMO E1 inhibitors (e.g., TAK-981) covalently binding UBA2 (Cys173), with combination (proteasome/demethylation inhibitors) and emerging (proteolysis-targeting chimera (proteolysis-targeting chimera (PROTAC)/targeted delivery) approaches. Optimization: Paired challenges (narrow therapeutic window, acquired resistance, patient stratification) and corresponding solutions (tissue-targeted delivery, next-generation inhibitors, multidimensional biomarkers)

The in-depth understanding of UBA2’s role in disease pathogenesis is driving its translation from basic research discoveries to clinical utility. This process is primarily reflected in two interrelated dimensions: First, developing UBA2 as a biomarker for disease diagnosis, prognosis assessment, and treatment response prediction; second, developing novel therapeutic interventions targeting UBA2 and its functional complex. The multifaceted clinical translation pathway of UBA2, encompassing its utility as a biomarker, its role as a therapeutic target, and the associated challenges, is illustrated in Fig. 6.

### UBA2 as a diagnostic and prognostic biomarker

UBA2 expression levels, genetic variants, or autoantibody status demonstrate significant biomarker potential in various diseases, with applications spanning oncology, autoimmune diseases, and genetic disorders.

In oncology, substantial clinical evidence indicates that high UBA2 expression is closely associated with poor patient prognosis. Studies indicate that UBA2 is an independent poor prognostic factor for overall survival and progression-free survival in multiple solid tumors, including HCC [3], glioma [49], LUAD [46], GC [43], ccRCC [59], and OSCC [71]. Its value extends beyond mere prognosis prediction to treatment response prediction. In LUAD, a prognostic signature model developed based on a set of SUMO pathway-related genes, including UBA2, not only predicts patient survival but also effectively distinguishes their sensitivity to adjuvant chemotherapy and potential features of the immunotherapy microenvironment, providing a molecular basis for personalized treatment decisions [46]. In NSCLC, a two-gene signature based on UBA2 (SAE2) and SAE1 also demonstrates robust prognostic predictive ability [47]. Particularly noteworthy is the finding that MYC-driven tumors exhibit a “synthetic lethal” effect with SUMOylation inhibition. This makes the MYC activation status a predictive biomarker for screening patients most likely to benefit from SUMO E1 inhibitor therapy, preliminarily realizing “biomarker-guided precision therapy“ [67, 123–125].

In autoimmune diseases, UBA2 (as the SAE2 subunit of the SUMO E1 enzyme) is the direct target of DM-specific autoantibodies. Anti-SAE antibodies have been identified as highly specific serological markers for DM. Their detection not only aids in the diagnosis and clinical subtyping of the disease, but studies also indicate that antibody positivity is associated with an increased risk of skin adverse reactions to hydroxychloroquine in patients, providing clear clinical warning and guidance value [74, 93]. However, clinical interpretation requires caution, as these antibodies may also appear in other autoimmune contexts, emphasizing the need for comprehensive judgment based on clinical manifestations [94].

In the molecular diagnosis of hereditary diseases, UBA2 gene testing has become essential for definitive diagnosis. For patients with complex phenotypes, such as SHFM and aplasia cutis congenita, identifying pathogenic UBA2 variants (for example, frameshift and missense mutations) through techniques, such as exome sequencing, can provide a definitive genetic diagnosis for diseases, such as ACCES. This molecular diagnosis offers patients a clear etiological explanation and is the cornerstone for accurate genetic counseling, recurrence risk assessment, and future prenatal diagnosis [4, 75, 76, 79].

### Therapeutic strategies targeting uba2 and progress

Given UBA2’s pivotal role in the SUMOylation pathway, developing therapeutic strategies against it has become a highly promising direction, with inhibition of its enzymatic activity representing the most suitable approach.

#### SUMO E1 small molecule inhibitors

The most advanced current strategy is developing small-molecule covalent inhibitors targeting the SUMO E1 activating enzyme. ML-792 and TAK-981 (Subasumstat) are two representative drugs. They irreversibly bind the catalytic cysteine (Cys173) of the UBA2 subunit, blocking SUMO activation and inhibiting cellular SUMOylation [124, 126, 127]. Preclinical studies have revealed broad anti-tumor potential in various models. In hematological malignancies, such as MYC-driven high-risk B-cell lymphoma, MM, AML, and MCL models, SUMO E1 inhibitors have been shown to inhibit tumor cell proliferation and to induce cell death in preclinical models [62, 64, 67, 68]. In solid tumors, these agents have shown significant efficacy in preclinical studies against breast cancer, LUAD, and OC [53, 54, 60]. They exhibit dual mechanism of action: On one hand, they cause mitotic catastrophe and apoptosis in tumor cells; on the other hand, SUMO inhibition activates type I interferon (IFN) signaling, remodels the tumor immune microenvironment, and improves anti-tumor immune responses, exhibiting a unique “immune-sensitizing” effect [67, 128]. Moreover, this immunomodulatory function suggests potential for treating autoimmune diseases. In a mouse model of rheumatoid arthritis, inhibiting UBA2 effectively alleviated synovial inflammation and joint destruction [90].

#### Combination therapy strategies

Combination strategies are widely investigated to enhance efficacy, overcome resistance, or widen the therapeutic window. These approaches are generally designed to exploit synthetic lethality or to resensitize resistant cells. For instance, simultaneous inhibition of the SUMOylation and proteasome pathways can overwhelm cellular protein homeostasis and induce synergistic stress responses; combining the SUMO E1 inhibitor TAK-981 with the proteasome inhibitor carfilzomib effectively reverses proteasome inhibitor resistance in multiple myeloma models [64]. Additionally, co-targeting SUMOylation and epigenetic regulation may produce synthetic lethal effects in certain hematologic malignancies; in acute myeloid leukemia models, SUMO E1 inhibitors combined with the demethylating agent 5-azacytidine exhibit synergistic anti-leukemic activity [62]. Furthermore, inhibiting SUMOylation can lower the threshold for chemotherapy-induced cell death by modulating DNA damage repair and stress responses; accordingly, UBA2 inhibition sensitizes ovarian cancer and laryngeal squamous cell carcinoma cells to cisplatin treatment [60, 70].

#### Other targeting strategies

Beyond small molecule inhibitors, other innovative targeting strategies are being explored. For instance, gene therapy approaches have been proposed based on fusing the functional domain of UBA2 with the antiviral protein APOBEC3G; this strategy aims to resist degradation by the HIV Vif protein, offering a new concept for anti-HIV therapy [129]. Additionally, nucleic acid-based strategies such as antisense oligonucleotides (ASOs) have been investigated; designing ASOs that specifically target UBA2 mRNA could, in theory, downregulate its expression, and preliminary cytotoxicity studies have explored this approach [130]. Furthermore, targeting oncogenic fusion proteins represents a precision medicine strategy; developing specific blockers or degraders against fusion genes such as UBA2-WTIP could lead to more tailored therapeutic interventions [61].

### 3.3 Challenges in clinical translation and countermeasures

Despite the promising prospects for targeting UBA2, its successful translation into safe and effective clinical therapies still faces numerous serious challenges, primarily in three aspects: Treatment safety, drug resistance, and precision application.

#### Therapeutic Window and Toxicity

This is the core challenge. SUMOylation is crucial for normal embryonic development and maintenance of basic cellular functions. Animal models demonstrate that transient inhibition of SUMOylation during pregnancy leads to severe developmental defects, including craniofacial malformations and syndactyly in offspring [22], which corroborates the phenotypes of various congenital developmental syndromes in humans caused by UBA2 haploinsufficiency [4]. Consequently, systemic, long-term inhibition of SUMOylation may cause unpredictable developmental toxicity, hematological toxicity, or other tissue-specific side effects. Future directions to address this challenge include developing tissue-specific delivery systems (for instance, nanocarriers, and antibody-drug conjugates) to selectively enrich drugs at the disease site; optimizing intermittent dosing regimens to reduce continuous exposure risk; and developing non-covalent, allosteric, or inhibitors selective for specific SUMO paralogs (SUMO1 versus SUMO2/3), aiming to achieve therapeutic goals while preserving some physiological SUMOylation functions.

#### Drug Resistance Issues

As with almost all targeted drugs, resistance eventually emerges. Specific point mutations have been identified in the UBA2 protein (for instance, S95N and M97T) that can confer resistance to ML-792 in tumor cells [124]. This highlights the need for further research into the molecular mechanisms of resistance development and the development of next-generation inhibitors capable of overcoming common mutations. Simultaneously, developing rational combination therapy strategies from the outset of treatment (for example, combining with proteasome inhibitors, demethylating agents, or immune checkpoint inhibitors) is an important clinical strategy to prevent or delay resistance [62, 64].

#### Patient Stratification and Biomarker Optimization

Although MYC activation status is a promising predictive marker, a more sophisticated and dynamic biomarker system is required to select the beneficiary population. Future research should integrate panoramic data from genomics, transcriptomics, proteomics, and especially SUMOylomics, to develop multidimensional predictive models [46, 52]. Furthermore, current preclinical data indicate that SUMO inhibitors are more effective in hematological malignancies. However, their tissue penetration, pharmacokinetic characteristics, and efficacy in solid tumors remain to be validated through carefully designed clinical trials. Addressing these challenges is critical to translating the scientific potential of targeting UBA2 into tangible clinical benefits.

In summary, the clinical translation of UBA2 follows a dual-track pattern from “diagnostic tool” to “therapeutic target.” As a biomarker, its expression levels, genetic variants, and autoantibody status demonstrate clear value in tumor prognosis stratification, hereditary syndrome diagnosis, and autoimmune disease subtyping. As a therapeutic target, covalent inhibition of its catalytic activity, exemplified by SUMO E1 inhibitors, such as TAK-981, represents the current mainstream strategy. This strategy combines direct anti-tumor and immunomodulatory dual mechanisms, with the potential to overcome resistance in combination therapies. However, the translational path faces core challenges, including a narrow therapeutic window, the emergence of drug resistance, and the need for precise patient stratification. Future breakthroughs will rely on the synergistic development of targeted delivery systems, next-generation inhibitors, and multidimensional biomarker systems. The complete translational logic from basic mechanisms to clinical applications and from strategies to challenges is systematically summarized in this article (Table 2).

**Table 2:** Clinical Translation of UBA2: Applications, Strategies, and Challenges

Translation Direction	Specific Application/Strategy	Core Mechanism/Rationale	Key Findings and Representative Agents	Clinical Significance and Value	Refs.
As a Biomarker	Prognostic Marker (Pan-cancer)	UBA2 is overexpressed in multiple malignancies; its level positively correlates with disease progression and aggressiveness.	Studies in HCC, glioma, LUAD, GC, RCC, and OSCC confirm that high UBA2 expression is an independent poor prognostic factor.	Provides a universal molecular tool for risk stratification and prognosis assessment in multiple solid tumors.	[3, 43, 46, 49, 59, 71]
	Predicting Treatment Response	SUMOylation pathway activity affects cell sensitivity to chemotherapy and immunotherapy.	Prognostic models based on SUMO pathway genes (including UBA2), e.g., SUMOPS in LUAD, can distinguish patients sensitive to chemotherapy and those with a potentially favorable immune microenvironment.	Advances tumor treatment from “one-size-fits-all” towards individualized choices (chemotherapy vs. immunotherapy), enabling more precise clinical decisions.	[46, 47]
	Predicting Targeted Therapy Sensitivity	MYC-driven tumors are functionally dependent on intact SUMOylation, exhibiting a synthetic lethal effect.	MYC activation status can serve as a predictive biomarker to screen for the patient population most sensitive to SUMO E1 inhibitors.	Lays the foundation for conducting “biomarker-enriched” precision clinical trials, potentially improving drug development success rates.	[67, 123, 124]
	Serological Diagnostic Marker (Dermatomyositis)	UBA2 (SAE2 subunit) is the target of autoimmune attack, generating highly specific autoantibodies.	Anti-SAE antibodies have high specificity for DM diagnosis and certain predictive value (e.g., hydroxychloroquine adverse reaction risk).	Provides an important serological diagnostic and subtyping tool for DM, with potential value in guiding clinical medication.	[74, 93, 94]
	Molecular Diagnostic Marker (Genetic Syndromes)	UBA2 loss-of-function variants are the direct genetic cause of a series of congenital developmental malformations.	Pathogenic UBA2 variants can be identified in patients with syndromes like ACCES and SHFM via techniques such as exome sequencing.	Provides definitive molecular diagnosis for affected families, crucial for precise genetic counseling, risk assessment, and prenatal diagnosis.	[4, 75, 76, 79]
As a Therapeutic Target	SUMO E1 Small Molecule Inhibitors	Covalently and irreversibly bind the catalytic cysteine (Cys173) of UBA2, globally inhibiting SUMO activation.	Representative agents: ML-792, TAK-981 (Subasumstat). Show dual mechanisms in preclinical models: 1. Direct anti-tumor: Induces mitotic catastrophe and apoptosis, effective against MYC-high, NOTCH1-activated tumors and various hematological malignancies. 2. Immunomodulation: Activates type I interferon signaling, remodels the tumor immune microenvironment, has “immune-sensitizing” potential. 3. Expanded indications: Also shows therapeutic potential in rheumatoid arthritis models.	Represents a novel class of therapeutics with a new mechanism of action, directly killing tumor cells while modulating the tumor microenvironment, offering new strategies for cancer and autoimmune disease treatment.	[54, 67, 68, 90, 123, 124, 126, 128]
	Combination Therapy Strategies	Simultaneous inhibition of SUMOylation and other key pathways yields synergistic effects to enhance efficacy or overcome resistance.	1. Combination with proteasome inhibitors (e.g., TAK-981 + carfilzomib) synergistically overcomes resistance in MM preclinical models. 2. Combination with demethylating agents (e.g., with 5-azacitidine) enhances efficacy in AML models. 3. Combination with chemotherapeutic agents sensitizes OC, laryngeal cancer, etc., to cisplatin.	Provides effective combinatorial approaches to overcome the limitations of existing therapies (especially resistance), a key direction for future clinical development.	[60, 62, 64, 70]
	Other Emerging Targeting Strategies	Intervention utilizing specific biological properties or genetic abnormalities of UBA2.	1. Targeting oncogenic fusion genes: Developing inhibitors against specific fusion genes like UBA2-WTIP. 2. Gene therapy concept: Utilizing UBA2 domains to stabilize antiviral proteins (e.g., anti-HIV APOBEC3G). 3. Nucleic acid drugs: Designing antisense oligonucleotides targeting UBA2 mRNA.	Demonstrates the diverse possibilities of targeting UBA2, providing early proof-of-concept for developing more precise or innovative future therapies.	[61, 129, 130]
Core Challenges	Therapeutic Window and Potential Toxicity	SUMOylation is crucial for normal embryonic development and maintaining basic cellular functions; systemic inhibition may cause severe side effects.	Animal studies show that inhibiting SUMOylation during pregnancy causes severe fetal malformations; human genetics confirms UBA2 haploinsufficiency causes multiple developmental syndromes.	The primary safety challenge. Future efforts should focus on developing tissue-targeted delivery systems, optimizing dosing regimens, or developing paralog/allosteric-selective inhibitors to widen the therapeutic window.	[4, 22]
	Acquired Resistance	Tumor cells can evade drug inhibition via target mutation, among other ways.	Studies have identified specific point mutations in UBA2 protein (e.g., S95N, M97T) conferring acquired resistance to ML-792.	As with all targeted therapies, resistance is inevitable. Requires pre-emptive study of resistance mechanisms, developing next-generation drugs, or preventive rational combination strategies.	[124]
	Precise Patient Stratification	A more complex predictive system beyond single biomarkers (e.g., MYC) is needed to screen the optimal beneficiary population.	Current predictive markers remain imperfect; data on SUMO inhibitor penetration and efficacy in solid tumors are still insufficient compared to hematological malignancies.	Key to future successful clinical translation. Requires integrating multi-omics data (e.g., SUMOylome) to build more precise predictive models and carefully designing clinical trials for solid tumors.	[46, 52]

## Future perspectives

While this review has consolidated UBA2’s central role in health and disease, several “unknowns” specific to this gatekeeper warrant in-depth exploration to propel the field from descriptive association to mechanistic understanding and precision intervention.

### Deciphering the structural basis and pathological implications of sumo paralog selectivity

Biochemical data indicate that UBA2/SAE1 exhibits distinct affinities for different SUMO paralogs (SUMO1, SUMO2, and SUMO3) [11]. However, the structural dynamics governing this selectivity remain completely unresolved—no high-resolution structure of the UBA2/SAE1 complex bound to SUMO2 or SUMO3 has been reported to date. Do disease-associated mutations in UBA2 alter the activation ratio of SUMO1 versus SUMO2/3, leading to paralog-specific pathological outcomes? Future cryo-EM studies capturing UBA2/SAE1 in complex with different SUMO paralogs and ATP analogs, combined with paralog-selective SUMOylomics [131], could reveal whether shifting this balance represents a viable therapeutic strategy for diseases where specific SUMO paralogs are dysregulated.

### The paradox of haploinsufficiency versus oncogenic overexpression: unveiling non-catalytic functions

UBA2 presents a striking biological paradox: germline loss-of-function causes developmental malformations [4, 22, 75–78], while somatic overexpression drives cancer progression [3, 29, 32]. Does UBA2 possess functions beyond its catalytic E1 activity? Emerging evidence suggests that the SUMOylation machinery, including UBA2, participates in chromatin silencing and maintenance of genomic integrity independent of its enzymatic activity on soluble substrates [27, 121]. Whether UBA2 itself acts as a platform to assemble DNA repair or transcriptional complexes in a SUMO-activation-independent manner is unknown. Answering this could explain why UBA2 haploinsufficiency is non-redundant and suggests that separating its catalytic from non-catalytic functions might yield safer therapeutic windows.

### Mapping the spatiotemporal dynamics of UBA2 Activity in single cells

Current knowledge presents a static snapshot of UBA2 expression, yet its activity is dynamically regulated by redox state (via reversible disulfide bond formation with Ubc9) [16] and by auto-SUMOylation feedback loops at its C-terminus [12, 13]. How does the UBA2-Ubc9 redox switch respond to physiological versus pathological reactive oxygen species levels in real-time? Does auto-SUMOylation of UBA2 create a binding platform for specific reader proteins that alter its substrate specificity or subcellular localization? Developing genetically encoded FRET-based biosensors for UBA2 activity—analogous to those for kinases—would allow live-cell imaging of SUMOylation capacity, revealing how transient UBA2 inhibition or hyperactivation in specific compartments contributes to diseases such as ischemia-reperfusion injury [116] or neurodegeneration [105–107].

### Convergent evolution of pathogen targeting and the immune-sensitizing mechanism

Strikingly, HIV- [5], Shigella [107], and adenovirus [18] have all independently evolved mechanisms to disable the UBA2/SAE1 complex (Fig. 5). This convergent evolution underscores a critical but poorly defined “Achilles’ heel” in host immunity. What makes UBA2/SAE1 such a favored target? Is it the complex’s surface accessibility, its centrality in the type I interferon pathway [128], or its regulation of a specific set of antiviral restriction factors? Pharmacological SUMO E1 inhibition not only directly kills tumor cells but also activates interferon signaling and remodels the tumor immune microenvironment [67, 128]. Yet the precise molecular link between UBA2 inhibition and interferon induction—whether through cytosolic DNA sensing, RIG-I-like receptors, or other pathways—remains elusive. Systematically mapping the pathogen-UBA2 interface and the innate immune circuits controlled by SUMOylation could identify conserved vulnerable epitopes, opening avenues for host-directed therapies that protect UBA2 function from viral degradation or cleavage, as well as for optimizing combination immunotherapies.

## Conclusion

UBA2, as the “gatekeeper” of the crucial post-translational modification pathway SUMOylation, exemplifies the modern biomedical research paradigm, which progresses from basic biochemical discovery to elucidation of disease mechanisms and then to clinical translation. This review reveals that functional dysregulation of UBA2 (overexpression or loss-of-function) is a common molecular node connecting multiple seemingly unrelated human diseases, including malignant tumors, developmental malformations, autoimmune diseases, and infections. In tumors, it is the “accelerator” driving oncogenic network activation and metabolic reprogramming [3, 29, 32]. In development, it is the “quality control inspector” ensuring precise embryonic morphogenesis [4, 22]. In immunity, it is the “tuner” regulating inflammatory balance, while also becoming a target for pathogens to evade host defense [5, 100]. This multidimensional, cross-disease hub role makes it a rare molecule possessing both powerful diagnostic/prognostic value and clear therapeutic targeting potential.

## Data Availability

This manuscript is a review article and does not contain any original datasets. All data and findings discussed herein are based on previously published studies, which are fully referenced. Readers can access the underlying data by consulting the referenced publications.

## References

[1] Johnson ES, Schwienhorst I, Dohmen RJ, Blobel G. The Ubiquitin-like Protein Smt3p Is Activated for Conjugation to Other Proteins by an Aos1p/Uba2p Heterodimer. EMBO J. 1997;16:5509–19. 10.1093/emboj/16.18.5509. 9312010 PMC1170183

[2] Desterro JM, Rodriguez MS, Kemp GD, Hay RT. Identification of the Enzyme Required for Activation of the Small Ubiquitin-like Protein SUMO-1. J Biol Chem. 1999;274:10618–24. 10.1074/jbc.274.15.10618. 10187858

[3] Chen H, Li H, He M, Lai Z, Huang L, Wen D, Shi M, Kan A. UBA2 SUMOylates NQO1 and Promotes the Proliferation of Hepatocellular Carcinoma by Modulating the MAPK Pathway. Cancer Sci. 2024;115:2998–3012. 10.1111/cas.16290. 39013843 PMC11462937

[4] Schnur RE, Yousaf S, Liu J, Chung WK, Rhodes L, Marble M, Zambrano RM, Sobreira N, Jayakar P, Pierpont ME, et al. UBA2 Variants Underlie a Recognizable Syndrome with Variable Aplasia Cutis Congenita and Ectrodactyly. Genet Med. 2021;23:1624–35. 10.1038/s41436-021-01182-1. 34040189 PMC8463496

[5] Mete B, Pekbilir E, Bilge BN, Georgiadou P, Çelik E, Sutlu T, Tabak F, Sahin U. Human Immunodeficiency Virus Type 1 Impairs Sumoylation. Life Sci Alliance. 2022;5:e202101103. 10.26508/lsa.202101103. 35181598 PMC8860096

[6] Gong L, Li B, Millas S, Yeh ET. Molecular Cloning and Characterization of Human AOS1 and UBA2, Components of the Sentrin-Activating Enzyme Complex. FEBS Lett. 1999;448:185–9. 10.1016/s0014-5793(99)00367-1. 10217437

[7] Lu X, Olsen SK, Capili AD, Cisar JS, Lima CD, Tan DS. Designed Semisynthetic Protein Inhibitors of Ub/Ubl E1 Activating Enzymes. J Am Chem Soc. 2010;132:1748–9. 10.1021/ja9088549. 20099854 PMC2830896

[8] Wang J, Taherbhoy AM, Hunt HW, Seyedin SN, Miller DW, Miller DJ, Huang DT, Schulman BA. Crystal Structure of UBA2(Ufd)-Ubc9: Insights into E1-E2 Interactions in Sumo Pathways. PLoS ONE. 2010;5:e15805. 10.1371/journal.pone.0015805. 21209884 PMC3012696

[9] Okuma T, Honda R, Ichikawa G, Tsumagari N, Yasuda H. In Vitro SUMO-1 Modification Requires Two Enzymatic Steps, E1 and E2. Biochem Biophys Res Commun. 1999;254:693–8. 10.1006/bbrc.1998.9995. 9920803

[10] Werner A, Moutty M-C, Möller U, Melchior F. Performing in Vitro Sumoylation Reactions Using Recombinant Enzymes. Methods Mol Biol. 2009;497:187–99. 10.1007/978-1-59745-566-4_12. 19107418

[11] Wiryawan H, Dan K, Etuale M, Shen Y, Liao J. Determination of SUMO1 and ATP Affinity for the SUMO E1by Quantitative FRET Technology. Biotechnol Bioeng. 2015;112:652–8. 10.1002/bit.25480. 25333792

[12] Truong K, Lee TD, Chen Y. Small Ubiquitin-like Modifier (SUMO) Modification of E1 Cys Domain Inhibits E1 Cys Domain Enzymatic Activity. J Biol Chem. 2012;287:15154–63. 10.1074/jbc.M112.353789. 22403398 PMC3346124

[13] Tang Z, Hecker CM, Scheschonka A, Betz H. Protein Interactions in the Sumoylation Cascade: Lessons from X-Ray Structures. FEBS J. 2008;275:3003–15. 10.1111/j.1742-4658.2008.06459.x. 18492068

[14] Importin α/β Mediates Nuclear Import of Individual SUMO E1 Subunits and of the Holo-Enzyme. Mol Biol Cell. 2024;35:cor1. 10.1091/mbc.E10-05-0461_cor. 38767651 PMC11238077

[15] Truong K, Lee TD, Li B, Chen Y. Sumoylation of SAE2 C Terminus Regulates SAE Nuclear Localization. J Biol Chem. 2012;287:42611–9. 10.1074/jbc.M112.420877. 23095757 PMC3522262

[16] Bossis G, Melchior F. Regulation of SUMOylation by Reversible Oxidation of SUMO Conjugating Enzymes. Mol Cell. 2006;21:349–57. 10.1016/j.molcel.2005.12.019. 16455490

[17] Aichem A, Sailer C, Ryu S, Catone N, Stankovic-Valentin N, Schmidtke G, Melchior F, Stengel F, Groettrup M. The Ubiquitin-like Modifier FAT10 Interferes with SUMO Activation. Nat Commun. 2019;10:4452. 10.1038/s41467-019-12430-z. 31575873 PMC6773726

[18] Boggio R, Passafaro A, Chiocca S, Targeting. SUMO E1 to Ubiquitin Ligases: A Viral Strategy to Counteract Sumoylation. J Biol Chem. 2007;282:15376–82. 10.1074/jbc.M700889200. 17392274

[19] Weber AR, Schuermann D, Schär P. Versatile Recombinant SUMOylation System for the Production of SUMO-Modified Protein. PLoS ONE. 2014;9:e102157. 10.1371/journal.pone.0102157. 25007328 PMC4090232

[20] Song Y, Liao J. Systematic Determinations of SUMOylation Activation Intermediates and Dynamics by a Sensitive and Quantitative FRET Assay. Mol Biosyst. 2012;8:1723–9. 10.1039/c2mb05465e. 22466055

[21] Wang J, Cai S, Chen Y. Mechanism of E1-E2 Interaction for the Inhibition of Ubl Adenylation. J Biol Chem. 2010;285:33457–62. 10.1074/jbc.M110.135582. 20682785 PMC2963334

[22] Mata-Garrido J, Zafferri I, Nordlinger A, Loe-Mie Y, Dejean A, Cossec J-C. Transient Pharmacological Inhibition of SUMOylation during Pregnancy Induces Craniofacial Malformations in Offspring Mice. Eur J Cell Biol. 2025;104:151480. 10.1016/j.ejcb.2025.151480. 39985830

[23] Eifler K, Cuijpers SAG, Willemstein E, Raaijmakers JA, El Atmioui D, Ovaa H, Medema RH, Vertegaal AC. .O. SUMO Targets the APC/C to Regulate Transition from Metaphase to Anaphase. Nat Commun. 2018;9:1119. 10.1038/s41467-018-03486-4. 29549242 PMC5856775

[24] Prudden J, Perry JJP, Arvai AS, Tainer JA, Boddy MN. Molecular Mimicry of SUMO Promotes DNA Repair. Nat Struct Mol Biol. 2009;16:509–16. 10.1038/nsmb.1582. 19363481 PMC2711901

[25] Heessen S, Masucci MG, Dantuma NP. The UBA2 Domain Functions as an Intrinsic Stabilization Signal That Protects Rad23 from Proteasomal Degradation. Mol Cell. 2005;18:225–35. 10.1016/j.molcel.2005.03.015. 15837425

[26] Pallava R, Bisher S, Brik A. Total Chemical Synthesis of RNF4 by Sequential Native Chemical Ligation: C-To-N Versus N-To-C Strategies. J Org Chem. 2026;91:2965–72. 10.1021/acs.joc.5c03224. 41636446 PMC12930501

[27] Yang BX, El Farran CA, Guo HC, Yu T, Fang HT, Wang HF, Schlesinger S, Seah YFS, Goh GYL, Neo SP, et al. Systematic Identification of Factors for Provirus Silencing in Embryonic Stem Cells. Cell. 2015;163:230–45. 10.1016/j.cell.2015.08.037. 26365490 PMC4686136

[28] Kalamarz ME, Paddibhatla I, Nadar C, Govind S. Sumoylation Is Tumor-Suppressive and Confers Proliferative Quiescence to Hematopoietic Progenitors in Drosophila Melanogaster Larvae. Biol Open. 2012;1:161–72. 10.1242/bio.2012043. 23213407 PMC3507282

[29] Zhang J, Ouyang F, Gao A, Zeng T, Li M, Li H, Zhou W, Gao Q, Tang X, Zhang Q, et al. ESM1 Enhances Fatty Acid Synthesis and Vascular Mimicry in Ovarian Cancer by Utilizing the PKM2-Dependent Warburg Effect within the Hypoxic Tumor Microenvironment. Mol Cancer. 2024;23. 10.1186/s12943-024-02009-8. PMC11077861 38720298

[30] Xu D, Bi J, Guan Y, Luo X, Chen X, Lv Y, Jin Y. Effects of the E1 Activating Enzyme UBA2 on Porcine Oocyte Maturation, Apoptosis, and Embryonic Development in Vitro. Anim Sci J. 2021;92:e13548. 10.1111/asj.13548. 33835647

[31] Zhou Q, Yin Y, Yu M, Gao D, Sun J, Yang Z, Weng J, Chen W, Atyah M, Shen Y, et al. GTPBP4 Promotes Hepatocellular Carcinoma Progression and Metastasis via the PKM2 Dependent Glucose Metabolism. Redox Biol. 2022;56:102458. 10.1016/j.redox.2022.102458. 36116159 PMC9483790

[32] Pan J, Wu S, Pan Q, Zhang Y, He L, Yao Q, Chen J, Li J, Xu Y. CHAC1 Blockade Suppresses Progression of Lung Adenocarcinoma by Interfering with Glucose Metabolism via Hijacking PKM2 Nuclear Translocation. Cell Death Dis. 2024;15:728. 10.1038/s41419-024-07114-6. 39368995 PMC11455913

[33] Tu J, Chen Y, Cai L, Xu C, Zhang Y, Chen Y, Zhang C, Zhao J, Cheng J, Xie H, et al. Functional Proteomics Study Reveals SUMOylation of TFII-I Is Involved in Liver Cancer Cell Proliferation. J Proteome Res. 2015;14:2385–97. 10.1021/acs.jproteome.5b00062. 25869096

[34] Yang H, Gao S, Chen J, Lou W. UBE2I Promotes Metastasis and Correlates with Poor Prognosis in Hepatocellular Carcinoma. Cancer Cell Int. 2020;20:234. 10.1186/s12935-020-01311-x. 32536822 PMC7291483

[35] Wong N, Chan KY-Y, Macgregor PF, Lai PB-S, Squire JA, Beheshti B, Albert M, Leung TW-T. Transcriptional Profiling Identifies Gene Expression Changes Associated with IFN-Alpha Tolerance in Hepatitis C-Related Hepatocellular Carcinoma Cells. Clin Cancer Res. 2005;11:1319–26. 15709204

[36] Qin Y, Bao H, Pan Y, Yin M, Liu Y, Wu S, Li H. SUMOylation Alterations Are Associated with Multidrug Resistance in Hepatocellular Carcinoma. Mol Med Rep. 2014;9:877–81. 10.3892/mmr.2014.1882. 24399357

[37] He P, Sun X, Cheng H-J, Zou Y-B, Wang Q, Zhou C-L, Liu W-Q, Hao Y-M, Meng X-W. UBA2 Promotes Proliferation of Colorectal Cancer. Mol Med Rep. 2018;18:5552–62. 10.3892/mmr.2018.9613. 30387828 PMC6236309

[38] Cheng H, Sun X, Li J, He P, Liu W, Meng X. Knockdown of Uba2 Inhibits Colorectal Cancer Cell Invasion and Migration through Downregulation of the Wnt/β-Catenin Signaling Pathway. J Cell Biochem. 2018;119:6914–25. 10.1002/jcb.26890. 29744931

[39] Cheng Y, Wang Y, Cheng Y, Yang Q, Zhang L, Li Z, Cheng J. FOXD3-Induced miR-133a Blocks Progression and Metastasis of Colorectal Cancer through Regulating UBA2. J Cancer. 2021;12:6145–54. 10.7150/jca.60647. 34539887 PMC8425194

[40] Guo S, Zhu K-X, Yu W-H, Wang T, Li S, Wang Y-X, Zhang C-C, Guo J-Q. SH3PXD2A-AS1/miR-330-5p/UBA2 ceRNA Network Mediates the Progression of Colorectal Cancer through Regulating the Activity of the Wnt/β-Catenin Signaling Pathway. Environ Toxicol. 2021;36:1969–80. 10.1002/tox.23038. 33073888

[41] Torres S, Garcia-Palmero I, Bartolomé RA, Fernandez-Aceñero MJ, Molina E, Calviño E, Segura MF, Casal JI. Combined miRNA Profiling and Proteomics Demonstrates That Different miRNAs Target a Common Set of Proteins to Promote Colorectal Cancer Metastasis. J Pathol. 2017;242:39–51. 10.1002/path.4874. 28054337

[42] Chai R, Yu X, Tu S, Zheng B. Depletion of UBA Protein 2-like Protein Inhibits Growth and Induces Apoptosis of Human Colorectal Carcinoma Cells. Tumour Biol. 2016;37:13225–35. 10.1007/s13277-016-5159-y. 27456362

[43] Li J, Sun X, He P, Liu W-Q, Zou Y-B, Wang Q, Meng X-W. Ubiquitin-like Modifier Activating Enzyme 2 Promotes Cell Migration and Invasion through Wnt/β-Catenin Signaling in Gastric Cancer. World J Gastroenterol. 2018;24:4773–86. 10.3748/wjg.v24.i42.4773. 30479464 PMC6235804

[44] Liu Z-L, Wang S-K, Pang L, Meng X-W. circXRCC5 Foster Gastric Cancer Growth and Metastasis by the HNRNPC/circXRCC5/miR-655-3p/RREB1/UBA2 Positive Feedback Loop. Cancer Gene Ther. 2022;29:1648–61. 10.1038/s41417-022-00482-1. 35661832

[45] Jiang B, Fan X, Zhang D, Liu H, Fan C. Identifying UBA2 as a Proliferation and Cell Cycle Regulator in Lung Cancer A549 Cells. J Cell Biochem. 2019;120:12752–61. 10.1002/jcb.28543. 30848500

[46] Yu L, Lin N, Ye Y, Zhuang H, Zou S, Song Y, Chen X, Wang Q. The Prognosis, Chemotherapy and Immunotherapy Efficacy of the SUMOylation Pathway Signature and the Role of UBA2 in Lung Adenocarcinoma. Aging. 2024;16:4378–95. 10.18632/aging.205594. 38407971 PMC10968705

[47] Sheng H, Hao Z, Zhu L, Zeng Y, He J. Construction and Validation of a Two-Gene Signature Based on SUMOylation Regulatory Genes in Non-Small Cell Lung Cancer Patients. BMC Cancer. 2022;22:572. 10.1186/s12885-022-09575-4. 35606717 PMC9125860

[48] Joseph NA, Chiou S-H, Lung Z, Yang C-L, Lin T-Y, Chang H-W, Sun HS, Gupta SK, Yen L, Wang S-D, et al. The Role of HGF-MET Pathway and CCDC66 cirRNA Expression in EGFR Resistance and Epithelial-to-Mesenchymal Transition of Lung Adenocarcinoma Cells. J Hematol Oncol. 2018;11:74. 10.1186/s13045-018-0557-9. 29855336 PMC5984410

[49] Ou Y, Luo H, Zhang Q, Du T, Liu R, Wang D, Chen J, Dong M, Wang Y, Yang Z, et al. UBA2 as a Prognostic Biomarker and Potential Therapeutic Target in Glioma. Front Biosci (Landmark Ed). 2024;29. 10.31083/j.fbl2904144. 38682183

[50] Cao S, Wang D, Wang P, Liu Y, Dong W, Ruan X, Liu L, Xue Y, E T, Lin H, et al. SUMOylation of RALY Promotes Vasculogenic Mimicry in Glioma Cells via the FOXD1/DKK1 Pathway. Cell Biol Toxicol. 2023;39:3323–40. 10.1007/s10565-023-09836-3. 37906341 PMC10693529

[51] Li X, Meng Y. SUMOylation Regulator-Related Molecules Can Be Used as Prognostic Biomarkers for Glioblastoma. Front Cell Dev Biol. 2021;9:658856. 10.3389/fcell.2021.658856. 33898460 PMC8063029

[52] Elbashir MK, Alanazi A, Mahmood MA. Decoding Multi-Omics Signatures in Lower-Grade Glioma Using Protein-Protein Interaction-Informed Graph Attention Networks and Ensemble Learning. Diagnostics (Basel). 2025;15:2894. 10.3390/diagnostics15222894. 41300918 PMC12651222

[53] Gu J, Huang L, Zhang Y. Monensin Inhibits Proliferation, Migration, and Promotes Apoptosis of Breast Cancer Cells via Downregulating UBA2. Drug Dev Res. 2020;81:745–53. 10.1002/ddr.21683. 32462716

[54] Licciardello MP, Müllner MK, Dürnberger G, Kerzendorfer C, Boidol B, Trefzer C, Sdelci S, Berg T, Penz T, Schuster M, et al. NOTCH1 Activation in Breast Cancer Confers Sensitivity to Inhibition of SUMOylation. Oncogene. 2015;34:3780–90. 10.1038/onc.2014.319. 25263445

[55] Maguire SL, Peck B, Wai PT, Campbell J, Barker H, Gulati A, Daley F, Vyse S, Huang P, Lord CJ, et al. Three-Dimensional Modelling Identifies Novel Genetic Dependencies Associated with Breast Cancer Progression in the Isogenic MCF10 Model. J Pathol. 2016;240:315–28. 10.1002/path.4778. 27512948 PMC5082563

[56] Du L, Fakih MG, Rosen ST, Chen Y. SUMOylation of E2F1 Regulates Expression of EZH2. Cancer Res. 2020;80:4212–23. 10.1158/0008-5472.CAN-20-1259. 32816857 PMC8741410

[57] Tuccilli C, Baldini E, Sorrenti S, Di Gioia C, Bosco D, Ascoli V, Mian C, Barollo S, Rendina R, Coccaro C, et al. Papillary thyroid cancer is characterized by altered expression of genes involved in the sumoylation process. J Biol Regul Homeost Agents. 2015;29:655–62. 26403403

[58] Du X, Shi J. UBA2 Promotes the Progression of Renal Cell Carcinoma by Suppressing the P53 Signaling. Ir J Med Sci. 2022;191:1555–60. 10.1007/s11845-021-02763-4. 34467471

[59] Zhang G, Zou J, Shi J, Qian B, Qiu K, Liu Q, Xie T, He Z, Xu H, Liao Y, et al. Knockdown of Ubiquitin-like Modifier-Activating Enzyme 2 Promotes Apoptosis of Clear Cell Renal Cell Carcinoma Cells. Cell Death Dis. 2021;12:1067. 10.1038/s41419-021-04347-7. 34753901 PMC8578554

[60] Opławski M, Średnicka A, Niewiadomska E, Boroń D, Januszyk P, Grabarek BO. Clinical and Molecular Evaluation of Patients with Ovarian Cancer in the Context of Drug Resistance to Chemotherapy. Front Oncol. 2022;12:954008. 10.3389/fonc.2022.954008. 35992817 PMC9389532

[61] Lu X, Zhuang H, Yu Q, Zhang X, Wu Z, Zhang L, Xu Y, Wu B, Yang L, Ma A, et al. Identification of the UBA2-WTIP Fusion Gene in Acute Myeloid Leukemia. Exp Cell Res. 2018;371:409–16. 10.1016/j.yexcr.2018.08.035. 30179602

[62] Ma L, Zhang K, Zhang Z, Wang C, Ma M, Liu Y, Zhao Y, Gong Z, Liu N, Wei M, et al. USP5 Inhibition Enables Potential Therapy for t(8;21) AML through Ubiquitin-Mediated AML1-ETO Degradation in Patient-Derived Xenografts. Sci Transl Med. 2025;17:eadt9100. 10.1126/scitranslmed.adt9100. 40991730

[63] Bang B, Eisfeldt J, Barbany G, Harila-Saari A, Heyman M, Zachariadis V, Taylan F, Nordgren A. A Somatic UBA2 Variant Preceded ETV6-RUNX1 in the Concordant BCP-ALL of Monozygotic Twins. Blood Adv. 2022;6:2275–89. 10.1182/bloodadvances.2021005703. 34982829 PMC9006272

[64] Heynen GJJE, Baumgartner F, Heider M, Patra U, Holz M, Braune J, Kaiser M, Schäffer I, Bamopoulos SA, Ramberger E, et al. SUMOylation Inhibition Overcomes Proteasome Inhibitor Resistance in Multiple Myeloma. Blood Adv. 2023;7:469–81. 10.1182/bloodadvances.2022007875. 35917568 PMC9979771

[65] Zhao F, Che F. Integrating Single-Cell and Bulk RNA Profiles to Uncover Glutamine Metabolism’s Role in Prognosis and Immune Dynamics in Multiple Myeloma. BMC Cancer. 2025;25:887. 10.1186/s12885-025-14239-0. 40389878 PMC12087063

[66] Wang S, Juan J, Zhang Z, Du Y, Xu Y, Tong J, Cao B, Moran MF, Zeng Y, Mao X. Inhibition of the Deubiquitinase USP5 Leads to C-Maf Protein Degradation and Myeloma Cell Apoptosis. Cell Death Dis. 2017;8:e3058. 10.1038/cddis.2017.450. 28933784 PMC5636991

[67] Demel UM, Wirth M, Yousefian S, Zhang L, Isaakidis K, Dönig J, Böger M, Singh N, Köse H, Haas S, et al. Small-Molecule SUMO Inhibition for Biomarker-Informed B-Cell Lymphoma Therapy. Haematologica. 2023;108:555–67. 10.3324/haematol.2022.280995. 36134453 PMC9890013

[68] Hanel W, Lata P, Youssef Y, Tran H, Tsyba L, Sehgal L, Blaser BW, Huszar D, Helmig-Mason J, Zhang L, et al. A Sumoylation Program Is Essential for Maintaining the Mitotic Fidelity in Proliferating Mantle Cell Lymphoma Cells. Exp Hematol Oncol. 2022;11:40. 10.1186/s40164-022-00293-y. 35831896 PMC9277803

[69] Matsumoto Y, Tsukamoto T, Chinen Y, Shimura Y, Sasaki N, Nagoshi H, Sato R, Adachi H, Nakano M, Horiike S, et al. Detection of Novel and Recurrent Conjoined Genes in Non-Hodgkin B-Cell Lymphoma. J Clin Exp Hematop. 2021;61:71–7. 10.3960/jslrt.20033. 33883344 PMC8265495

[70] Yang L, Yan B, Qu L, Ren J, Li Q, Wang J, Kan X, Liu M, Wang Y, Sun Y, et al. IGF2BP3 Regulates TMA7-Mediated Autophagy and Cisplatin Resistance in Laryngeal Cancer via m6A RNA Methylation. Int J Biol Sci. 2023;19:1382–400. 10.7150/ijbs.80921. 37056932 PMC10086756

[71] Meng Y, Li X. Expression and Prognosis Analysis of SUMOylation Regulators in Oral Squamous Cell Carcinoma Based on High-Throughput Sequencing. Front Genet. 2021;12:671392. 10.3389/fgene.2021.671392. 34267779 PMC8277238

[72] Feng X, Matsuo K, Zhang T, Hu Y, Mays AC, Browne JD, Zhou X, Sullivan CA. MicroRNA Profiling and Target Genes Related to Metastasis of Salivary Adenoid Cystic Carcinoma. Anticancer Res. 2017;37:3473–81. 10.21873/anticanres.11715. 28668836

[73] Kumar P, Kumawat RK, Uttam V, Behera A, Rani M, Singh N, Barwal TS, Sharma U, Jain A. The Imminent Role of microRNAs in Salivary Adenoid Cystic Carcinoma. Transl Oncol. 2023;27:101573. 10.1016/j.tranon.2022.101573. 36335706 PMC9646983

[74] Tarricone E, Ghirardello A, Rampudda M, Bassi N, Punzi L, Doria A. Anti-SAE Antibodies in Autoimmune Myositis: Identification by Unlabelled Protein Immunoprecipitation in an Italian Patient Cohort. J Immunol Methods. 2012;384:128–34. 10.1016/j.jim.2012.07.019. 22884621

[75] Aerden M, Bauters M, Van Den Bogaert K, Vermeesch JR, Holvoet M, Plasschaert F, Devriendt K. Genotype-Phenotype Correlations of UBA2 Mutations in Patients with Ectrodactyly. Eur J Med Genet. 2020;63:104009. 10.1016/j.ejmg.2020.104009. 32758660

[76] Parveen A, Tariq M, Khan SA, Kakar N, Arif A, Wasif NA. Novel Frameshift Variant in UBA2 Causing Split-Hand/Foot Malformations in a Pakistani Family. Hum Genome Var. 2023;10:16. 10.1038/s41439-023-00242-z. 37221169 PMC10206101

[77] Yamoto K, Saitsu H, Nishimura G, Kosaki R, Takayama S, Haga N, Tonoki H, Okumura A, Horii E, Okamoto N, et al. Comprehensive Clinical and Molecular Studies in Split-Hand/Foot Malformation: Identification of Two Plausible Candidate Genes (LRP6 and UBA2). Eur J Hum Genet. 2019;27:1845–57. 10.1038/s41431-019-0473-7. 31332306 PMC6871171

[78] Carter TC, Kay DM, Pangilinan F, Almli LM, Jenkins MM, Blue EE, Sok P, White JJ, Cunniff CM, Agopian AJ, et al. Exome Sequencing to Identify Novel Susceptibility Genes for Nonsyndromic Split-Hand/Ft Malformation: A Report From the National Birth Defects Prevention Study. Birth Defects Res. 2025;117:e2472. 10.1002/bdr2.2472. 40304391 PMC12818101

[79] Liaqat K, Felipe K, Treat K, McPheron M, Weaver DD, Vetrini F, Conboy E. Uncovering a Diagnosis Through Reanalysis of UBA2 Variants in a Patient with Syndactyly, Polydactyly, and Aplasia Cutis Congenita: A Short Report and a Review of the Literature. Genet Test Mol Biomarkers. 2025;29:120–8. 10.1089/gtmb.2025.0042. 40249340

[80] Sezer A, Özdemir Z, Özkan E, Çetinkaya S. Atypical Presentation of ACCES Syndrome Resembling Dominant Spondyloepiphyseal Dysplasia Tarda. Am J Med Genet A. 2024;194:e63852. 10.1002/ajmg.a.63852. 39149811

[81] Venegas-Vega C, Nieto-Martínez K, Martínez-Herrera A, Gómez-Laguna L, Berumen J, Cervantes A, Kofman S, Fernández-Ramírez F. q13.11 Microdeletion Concomitant with Ins(2;19)(P25.3;Q13.1q13.4)Dn in a Boy: Potential Role of UBA2 in the Associated Phenotype. Mol Cytogenet. 2014;7:19. 10.1186/s13039-014-0061-z. 25516771 PMC4266984

[82] Melo JB, Estevinho A, Saraiva J, Ramos L, Carreira IM. Cutis Aplasia as a Clinical Hallmark for the Syndrome Associated with 19q13.11 Deletion: The Possible Role for UBA2 Gene. Mol Cytogenet. 2015;8:21. 10.1186/s13039-015-0123-x. 25883683 PMC4399573

[83] Abe KT, Rizzo IMPO, Coelho ALV, Sakai N, Carvalho DR, Speck-Martins CE. 19q13.11 Microdeletion: Clinical Features Overlapping Ectrodactyly Ectodermal Dysplasia-Clefting Syndrome Phenotype. Clin Case Rep. 2018;6:1300–7. 10.1002/ccr3.1600. 29988626 PMC6028370

[84] Hardcastle A, Berry AM, Campbell IM, Zhao X, Liu P, Gerard AE, Rosenfeld JA, Sisoudiya SD, Hernandez-Garcia A, Loddo S, et al. Identifying Phenotypic Expansions for Congenital Diaphragmatic Hernia plus (CDH+) Using DECIPHER Data. Am J Med Genet A. 2022;188:2958–68. 10.1002/ajmg.a.62919. 35904974 PMC9474674

[85] Marble M, Guillen Sacoto MJ, Chikarmane R, Gargiulo D, Juusola J. Missense Variant in UBA2 Associated with Aplasia Cutis Congenita, Duane Anomaly, Hip Dysplasia and Other Anomalies: A Possible New Disorder Involving the SUMOylation Pathway. Am J Med Genet A. 2017;173:758–61. 10.1002/ajmg.a.38078. 28110515

[86] Sun L, Huang Y, Zhao S, Zhao J, Yan Z, Guo Y, Lin M, Zhong W, Yin Y, Chen Z, et al. Deciphering the Mutational Signature of Congenital Limb Malformations. Mol Ther Nucleic Acids. 2021;24:961–70. 10.1016/j.omtn.2021.04.012. 34094714 PMC8141661

[87] Elsner J, Mensah MA, Holtgrewe M, Hertzberg J, Bigoni S, Busche A, Coutelier M, de Silva DC, Elçioglu N, Filges I, et al. Genome Sequencing in Families with Congenital Limb Malformations. Hum Genet. 2021;140:1229–39. 10.1007/s00439-021-02295-y. 34159400 PMC8263393

[88] Balachandran S, Prada-Medina CA, Mensah MA, Kakar N, Nagel I, Pozojevic J, Audain E, Hitz M-P, Kircher M, Sreenivasan VKA, et al. STIGMA: Single-Cell Tissue-Specific Gene Prioritization Using Machine Learning. Am J Hum Genet. 2024;111:338–49. 10.1016/j.ajhg.2023.12.011. 38228144 PMC10870135

[89] Netzer C, Bohlander SK, Rieger L, Müller S, Kohlhase J. Interaction of the Developmental Regulator SALL1 with UBE2I and SUMO-1. Biochem Biophys Res Commun. 2002;296:870–6. 10.1016/s0006-291x(02)02003-x. 12200128

[90] Wang C, Xiao Y, Lao M, Wang J, Xu S, Li R, Xu X, Kuang Y, Shi M, Zou Y, et al. Increased SUMO-Activating Enzyme SAE1/UBA2 Promotes Glycolysis and Pathogenic Behavior of Rheumatoid Fibroblast-like Synoviocytes. JCI Insight. 2020;5(e135935):135935. 10.1172/jci.insight.135935. 32938830 PMC7526534

[91] Wu Q, Jiang Y, You C. The SUMO Components in Rheumatoid Arthritis. Rheumatology (Oxford). 2022;61:4619–30. 10.1093/rheumatology/keac297. 35595244

[92] Tartar DM, Chung L, Fiorentino DF. Clinical Significance of Autoantibodies in Dermatomyositis and Systemic Sclerosis. Clin Dermatol. 2018;36:508–24. 10.1016/j.clindermatol.2018.04.008. 30047434

[93] Wolstencroft PW, Casciola-Rosen L, Fiorentino DF. Association Between Autoantibody Phenotype and Cutaneous Adverse Reactions to Hydroxychloroquine in Dermatomyositis. JAMA Dermatol. 2018;154:1199–203. 10.1001/jamadermatol.2018.2549. 30140893 PMC6233745

[94] Victor J, Zanardo L, Héron-Mermin D, Poursac N, Solé G, Bordes C, Duffau P. Retrospective analysis of anti-TIF1gamma, anti-NXP2 and anti-SAE1/2 antibodies carriers at Bordeaux university hospital from November 2014 to February 2017]. Rev Med Interne. 2019;40:70–81. 10.1016/j.revmed.2018.11.003. 30527961

[95] De Sadeleer LJ, De Langhe E, Bodart N, Vigneron A, Bossuyt X, Wuyts WA. Prevalence of Myositis-Specific Antibodies in Idiopathic Interstitial Pneumonias. Lung. 2018;196:329–33. 10.1007/s00408-018-0108-8. 29532165

[96] Mustfa SA, Singh M, Suhail A, Mohapatra G, Verma S, Chakravorty D, Rana S, Rampal R, Dhar A, Saha S, et al. SUMOylation Pathway Alteration Coupled with Downregulation of SUMO E2 Enzyme at Mucosal Epithelium Modulates Inflammation in Inflammatory Bowel Disease. Open Biol. 2017;7:170024. 10.1098/rsob.170024. 28659381 PMC5493774

[97] Jung J, Byeon I-JL, DeLucia M, Koharudin LMI, Ahn J, Gronenborn AM. Binding of HIV-1 Vpr Protein to the Human Homolog of the Yeast DNA Repair Protein RAD23 (hHR23A) Requires Its Xeroderma Pigmentosum Complementation Group C Binding (XPCB) Domain as Well as the Ubiquitin-Associated 2 (UBA2) Domain. J Biol Chem. 2014;289:2577–88. 10.1074/jbc.M113.534453. 24318982 PMC3908392

[98] Byeon I-JL, Calero G, Wu Y, Byeon CH, Jung J, DeLucia M, Zhou X, Weiss S, Ahn J, Hao C, et al. Structure of HIV-1 Vpr in Complex with the Human Nucleotide Excision Repair Protein hHR23A. Nat Commun. 2021;12:6864. 10.1038/s41467-021-27009-w. 34824204 PMC8617076

[99] Withers-Ward ES, Mueller TD, Chen IS, Feigon J. Biochemical and Structural Analysis of the Interaction between the UBA(2) Domain of the DNA Repair Protein HHR23A and HIV-1 Vpr. Biochemistry. 2000;39:14103–12. 10.1021/bi0017071. 11087358

[100] Lapaquette P, Fritah S, Lhocine N, Andrieux A, Nigro G, Mounier J, Sansonetti P, Dejean A. Shigella Entry Unveils a Calcium/Calpain-Dependent Mechanism for Inhibiting Sumoylation. Elife. 2017;6:e27444. 10.7554/eLife.27444. 29231810 PMC5745084

[101] Reiter K, Mukhopadhyay D, Zhang H, Boucher LE, Kumar N, Bosch J, Matunis MJ. Identification of Biochemically Distinct Properties of the Small Ubiquitin-Related Modifier (SUMO) Conjugation Pathway in Plasmodium Falciparum. J Biol Chem. 2013;288:27724–36. 10.1074/jbc.M113.498410. 23943616 PMC3784690

[102] Anyona SB, Cheng Q, Raballah E, Hurwitz I, Lambert CG, McMahon BH, Ouma C, Perkins DJ. Ingestion of Hemozoin by Peripheral Blood Mononuclear Cells Alters Temporal Gene Expression of Ubiquitination Processes. Biochem Biophys Rep. 2022;29:101207. 10.1016/j.bbrep.2022.101207. 35071802 PMC8761598

[103] Pereira RV, Gomes M, de Cabral S, Jannotti-Passos FJ, Rodrigues LK, de Castro-Borges V, Guerra-Sá W. Up-Regulation of SUMO E3 Ligases during Lung Schistosomula and Adult Worm Stages. Parasitol Res. 2014;113:2019–25. 10.1007/s00436-014-3841-9. 24802862

[104] Vélez N, Monteoliva L, Sánchez-Quitian Z-A, Amador-García A, García-Rodas R, Ceballos-Garzón A, Gil C, Escandón P, Zaragoza Ó, Parra-Giraldo C-M. The Combination of Iron and Copper Increases Pathogenicity and Induces Proteins Related to the Main Virulence Factors in Clinical Isolates of Cryptococcus Neoformans Var. Grubii. J Fungi (Basel). 2022;8:57. 10.3390/jof8010057. 35049997 PMC8778102

[105] Munari F, Bortot A, Assfalg M, D’Onofrio M. Alzheimer’s Disease-Associated Ubiquitin Mutant Ubb + 1: Properties of the Carboxy-Terminal Domain and Its Influence on Biomolecular Interactions. Int J Biol Macromol. 2018;108:24–31. 10.1016/j.ijbiomac.2017.11.121. 29175520

[106] Vavougios GD, Zarogiannis SG, Krogfelt KA, Gourgoulianis K, Mitsikostas DD, Hadjigeorgiou G. Novel Candidate Genes of the PARK7 Interactome as Mediators of Apoptosis and Acetylation in Multiple Sclerosis: An in Silico Analysis. Mult Scler Relat Disord. 2018;19:8–14. 10.1016/j.msard.2017.10.013. 29100048

[107] Chan HYE, Warrick JM, Andriola I, Merry D, Bonini NM. Genetic Modulation of Polyglutamine Toxicity by Protein Conjugation Pathways in Drosophila. Hum Mol Genet. 2002;11:2895–904. 10.1093/hmg/11.23.2895. 12393801

[108] Koh K, Park M, Bae ES, Duong V-A, Park J-M, Lee H, Lew H. UBA2 Activates Wnt/β-Catenin Signaling Pathway during Protection of R28 Retinal Precursor Cells from Hypoxia by Extracellular Vesicles Derived from Placental Mesenchymal Stem Cells. Stem Cell Res Ther. 2020;11:428. 10.1186/s13287-020-01943-w. 33008487 PMC7532108

[109] Loftus LT, Gala R, Yang T, Jessick VJ, Ashley MD, Ordonez AN, Thompson SJ, Simon RP, Meller R. Sumo-2/3-Ylation Following in Vitro Modeled Ischemia Is Reduced in Delayed Ischemic Tolerance. Brain Res. 2009;1272:71–80. 10.1016/j.brainres.2009.03.034. 19332039 PMC2774733

[110] Nie Q, Gong X, Gong L, Zhang L, Tang X, Wang L, Liu F, Fu J-L, Xiang J-W, Xiao Y, et al. Sodium Iodate-Induced Mouse Model of Age-Related Macular Degeneration Displayed Altered Expression Patterns of Sumoylation Enzymes E1, E2 and E3. Curr Mol Med. 2018;18:550–5. 10.2174/1566524019666190112101147. 30636606

[111] Nie Q, Wang L, Gong X, Xiang J-W, Xiao Y, Xie J, Yang L, Chen H, Gan Y, Chen Z, et al. Altered Expression Patterns of the Sumoylation Enzymes E1, E2 and E3 Are Associated with Glucose Oxidase- and UVA-Induced Cataractogenesis. Curr Mol Med. 2018;18:542–9. 10.2174/1566524019666190111152324. 30636603

[112] Nie Q, Xie J, Gong X, Luo Z, Wang L, Liu F, Xiang J-W, Xiao Y, Fu J-L, Liu Y, et al. Analysis of the Differential Expression Patterns of Sumoylation Enzymes E1, E2 and E3 in Ocular Cell Lines. Curr Mol Med. 2018;18:509–15. 10.2174/1566524019666190112143636. 30636610

[113] Nie Q, Yang L, Qing W, Xie J, Gong X, Chen H, Gan Y, Wang L, Xiang J-W, Xiao Y, et al. Differential Expression of Sumoylation Enzymes SAE1, U BA2, UBC9, PIAS1 and RanBP2 in Major Ocular Tissues of Mouse Eye. Curr Mol Med. 2018;18:376–82. 10.2174/1566524018666181116112208. 30479214

[114] Gong X, Nie Q, Xiao Y, Xiang J-W, Wang L, Liu F, Fu J-L, Liu Y, Yang L, Gan Y, et al. Localization Patterns of Sumoylation Enzymes E1, E2 and E3 in Ocular Cell Lines Predict Their Functional Importance. Curr Mol Med. 2018;18:516–22. 10.2174/1566524019666190112144436. 30636611

[115] Qi Y, Guan H, Liang X, Sun J, Yao W. Ang II Promotes SUMO2/3 Modification of RhoGDI1 Through Aos1 and Uba2 Subunits, and Then Regulates RhoGDI1 Stability and Cell Proliferation. Cardiovasc Drugs Ther. 2021;35:769–73. 10.1007/s10557-021-07173-3. 33891248

[116] Huang J, Xie P, Dong Y, An W. Inhibition of Drp1 SUMOylation by ALR Protects the Liver from Ischemia-Reperfusion Injury. Cell Death Differ. 2021;28:1174–92. 10.1038/s41418-020-00641-7. 33110216 PMC8027887

[117] Tomati V, Pesce E, Caci E, Sondo E, Scudieri P, Marini M, Amato F, Castaldo G, Ravazzolo R, Galietta LJV, et al. High-Throughput Screening Identifies FAU Protein as a Regulator of Mutant Cystic Fibrosis Transmembrane Conductance Regulator Channel. J Biol Chem. 2018;293:1203–17. 10.1074/jbc.M117.816595. 29158263 PMC5787799

[118] Romanenko AM, Kinoshita A, Wanibuchi H, Wei M, Zaparin WK, Vinnichenko WI, Vozianov AF, Fukushima S. Involvement of Ubiquitination and Sumoylation in Bladder Lesions Induced by Persistent Long-Term Low Dose Ionizing Radiation in Humans. J Urol. 2006;175:739–43. 10.1016/S0022-5347(05)00172-2. 16407042

[119] da Silva Z, Glanzner WG, Currin L, de Macedo MP, Gutierrez K, Guay V, Gonçalves PBD, Bordignon V. DNA Damage Induction Alters the Expression of Ubiquitin and SUMO Regulators in Preimplantation Stage Pig Embryos. Int J Mol Sci. 2022;23:9610. 10.3390/ijms23179610. 36077022 PMC9455980

[120] Kim CY, Bove J, Assmann SM. Overexpression of Wound-Responsive RNA-Binding Proteins Induces Leaf Senescence and Hypersensitive-like Cell Death. New Phytol. 2008;180:57–70. 10.1111/j.1469-8137.2008.02557.x. 18705666

[121] Poleshko A, Kossenkov AV, Shalginskikh N, Pecherskaya A, Einarson MB, Marie Skalka A, Katz RA. Human Factors and Pathways Essential for Mediating Epigenetic Gene Silencing. Epigenetics. 2014;9:1280–9. 10.4161/epi.32088. 25147916 PMC4169020

[122] Costa MW, Lee S, Furtado MB, Xin L, Sparrow DB, Martinez CG, Dunwoodie SL, Kurtenbach E, Mohun T, Rosenthal N, et al. Complex SUMO-1 Regulation of Cardiac Transcription Factor Nkx2-5. PLoS ONE. 2011;6:e24812. 10.1371/journal.pone.0024812. 21931855 PMC3171482

[123] Kessler JD, Kahle KT, Sun T, Meerbrey KL, Schlabach MR, Schmitt EM, Skinner SO, Xu Q, Li MZ, Hartman ZC, et al. A SUMOylation-Dependent Transcriptional Subprogram Is Required for Myc-Driven Tumorigenesis. Science. 2012;335:348–53. 10.1126/science.1212728. 22157079 PMC4059214

[124] He X, Riceberg J, Soucy T, Koenig E, Minissale J, Gallery M, Bernard H, Yang X, Liao H, Rabino C, et al. Probing the Roles of SUMOylation in Cancer Cell Biology by Using a Selective SAE Inhibitor. Nat Chem Biol. 2017;13:1164–71. 10.1038/nchembio.2463. 28892090

[125] Mattoscio D, Chiocca SSUMO. Pathway Components as Possible Cancer Biomarkers. Future Oncol. 2015;11:1599–610. 10.2217/fon.15.41. 26043214

[126] Lv Z, Yuan L, Atkison JH, Williams KM, Vega R, Sessions EH, Divlianska DB, Davies C, Chen Y, Olsen SK. Molecular Mechanism of a Covalent Allosteric Inhibitor of SUMO E1 Activating Enzyme. Nat Commun. 2018;9:5145. 10.1038/s41467-018-07015-1. 30514846 PMC6279746

[127] Li Y-J, Du L, Aldana-Masangkay G, Wang X, Urak R, Forman SJ, Rosen ST, Chen Y. Regulation of miR-34b/c-Targeted Gene Expression Program by SUMOylation. Nucleic Acids Res. 2018;46:7108–23. 10.1093/nar/gky484. 29893976 PMC6101486

[128] Kroonen JS, Vertegaal ACO. Targeting SUMO Signaling to Wrestle Cancer. Trends Cancer. 2021;7:496–510. 10.1016/j.trecan.2020.11.009. 33353838

[129] Li L, Liang D, Li J, Zhao RY. APOBEC3G-UBA2 Fusion as a Potential Strategy for Stable Expression of APOBEC3G and Inhibition of HIV-1 Replication. Retrovirology. 2008;5:72. 10.1186/1742-4690-5-72. 18680593 PMC2535603

[130] Popova KB, Penchovsky R. General and Specific Cytotoxicity of Chimeric Antisense Oligonucleotides in Bacterial Cells and Human Cell Lines. Antibiot (Basel). 2024;13:122. 10.3390/antibiotics13020122. PMC10885958 38391508

[131] Albuquerque CP, Yeung E, Ma S, Fu T, Corbett KD, Zhou HA. Chemical and Enzymatic Approach to Study Site-Specific Sumoylation. PLoS ONE. 2015;10:e0143810. 10.1371/journal.pone.0143810. 26633173 PMC4669148

